# Climate and hybridization shape stomatal trait evolution in *Populus*

**DOI:** 10.1111/nph.70706

**Published:** 2025-11-17

**Authors:** Michelle Zavala‐Paez, Stephen R. Keller, Jason Holliday, Matthew C. Fitzpatrick, Jill A. Hamilton

**Affiliations:** ^1^ Pennsylvania State University University Park PA 16803 USA; ^2^ University of Vermont Burlington VT 05405 USA; ^3^ Virginia Tech Blacksburg VA 24061 USA; ^4^ University of Maryland Center for Environmental Science Frostburg MD 21532 USA

**Keywords:** adaptation, ecophysiology, evolution, hybrids, introgression, *Populus*, trees

## Abstract

- Stomata play a critical role in regulating plant responses to climate. Where sister species differ in stomatal traits, interspecific gene flow can influence the evolutionary trajectory of trait variation, with consequences for adaptation.
- Leveraging six latitudinally distributed contact zones spanning the natural hybrid zone between *Populus trichocarpa × Populus balsamifera*, we used whole genome resequencing and replicate common garden experiments to test the role that interspecific gene flow and selection play in stomatal trait evolution.
- While species‐specific differences in the distribution of stomata persist between *P. balsamifera* and *P. trichocarpa*, hybrids on average resemble *P. trichocarpa*. Admixture mapping identified several candidate genes associated with stomatal trait variation in hybrids, including *TWIST*, a homolog of *SPEECHLESS* in *Arabidopsis*, which initiates stomatal development via asymmetric cell divisions. Geographic clines revealed candidate genes deviating from genome‐wide average patterns of introgression, suggesting restricted gene flow and the maintenance of adaptive differences. Climate associations, particularly with precipitation, indicated selection shapes local ancestry at candidate genes across contact zones.
- These results highlight the role of climate in shaping stomatal trait evolution in *Populus* and demonstrate how interspecific gene flow creates novel genetic combinations that may enhance adaptive potential in changing environments.

## Resumen

- Los estomas desempeñan un papel fundamental en la regulación de las respuestas de las plantas a cambios ambientales. Cuando especies cercanas difieren en sus rasgos estomáticos, el flujo génico interespecífico puede influir en la trayectoria evolutiva de la variación fenotípica y, por lo tanto, tener consecuencias para la adaptación.
- Aprovechando seis transectos distribuidos latitudinalmente a lo largo de la zona híbrida natural entre *Populus trichocarpa* y *P. balsamifera*, utilizamos datos de resecuenciación del genoma completo y experimentos de jardín común con clones replicados para evaluar el papel del flujo génico interespecífico y la selección en la evolución de los rasgos estomáticos.
- Aunque persistieron diferencias específicas entre especies en la distribución de los estomas entre las distintas superficies de hojas de *P. balsamifera* y *P. trichocarpa*, los híbridos, en promedio, se asemejaron más a *P. trichocarpa*. Estudios de asociación a nivel del genoma identificaron varios genes candidatos asociados con la variación de los rasgos estomáticos en los híbridos, incluido *TWIST*, un homólogo de *SPEECHLESS* en *Arabidopsis*, que inicia el desarrollo de los estomas mediante divisiones celulares asimétricas. Las clinas geográficas revelaron genes candidatos que se desviaron de los patrones promedio de introgresión a nivel genómico, lo que sugiere flujo génico restringido y el mantenimiento de diferencias adaptativas. Las asociaciones con el clima, particularmente con la precipitación, indicaron que la selección actúa en la ascendencia local en los genes candidatos a lo largo de los transectos.
- Estos resultados resaltan el papel del clima en la evolución de los rasgos estomáticos en *Populus* y demuestran cómo el flujo génico interespecífico puede generar nuevas combinaciones genéticas que aumentan el potencial adaptativo en ambientes cambiantes.

## Introduction

Hybridization is an important source of variation that enhances adaptive evolution by creating new recombinant genotypes and increasing standing genetic variation (Rieseberg & Wendel, 1993; Burke & Arnold, 2001). The movement of alleles from one species into the genetic background of another via repeated backcrossing can facilitate adaptive evolution (Hamilton & Aitken, 2013; Hamilton *et al*., 2013; Hamilton & Miller, 2016; Suarez‐Gonzalez *et al*., 2018a,b; Menon *et al*., 2021). This is particularly notable in plant species, where adaptive introgression has been associated with traits related to water use that are critical to maintaining physiological function under changing environmental conditions (Welch & Rieseberg, 2002; Campbell, 2004; Wu & Campbell, 2007; Whitney *et al*., 2010; Campbell & Wendlandt, 2013; Suarez‐Gonzalez *et al*., 2018a,b). Despite potential benefits from introgression, fine‐scale assessments of the genetic variation underlying adaptive trait variation and the role of extrinsic selection in introgression across different genomic backgrounds remain limited. This study aims to understand how natural selection shapes the movement of genetic variation across species boundaries, which is essential for predicting how recombinant genotypes may respond to climate change.

Stomatal traits are fundamental to photosynthesis, transpiration, and overall water balance, but also exhibit substantial variation across species (Hetherington & Woodward, 2003; Drake *et al*., 2013; Marek *et al*., 2022; Chen *et al*., 2024). These microscopic pores on the leaf surface regulate CO₂ uptake and water vapor loss, making them key targets of selection under changing environmental conditions (Chen *et al*., 2024). Stomata have evolved variation in size, density, and distribution across upper (adaxial) and lower (abaxial) leaf surfaces to optimize trade‐offs associated with carbon uptake, water‐use efficiency, and pathogen exposure across environments (Hetherington & Woodward, 2003; Dittberner *et al*., 2018; Chen *et al*., 2024). Intraspecific variation in stomatal traits is often structured along climatic gradients. In general, populations from warmer, drier, or more seasonally extreme environments tend to exhibit higher stomatal densities and maintain smaller pore sizes. This contrasts with populations from cooler or wet environments, which generally maintain fewer, large stomata and exhibit reduced conductance (Dittberner *et al*., 2018; Elfarargi *et al*., 2023; Cai *et al*., 2024; Pan *et al*., 2024). The distribution of stomata across leaf surfaces further influences these strategies, as amphistomy supports higher conductance and carbon gain in high‐light environments while hypostomy limits water loss and pathogen exposure in shaded, cooler, or arid conditions (Drake *et al*., 2019; Muir, 2019; Liu *et al*., 2024). However, despite growing evidence that stomatal traits evolve in response to climate, much less is known about how interspecific gene flow shapes stomatal evolution (but see Fetter *et al*., 2021; Fetter & Keller, 2023). Natural hybrid zones provide ideal systems to understand how varying genomic and environmental backgrounds shape the broad and fine‐scale evolutionary trajectory of stomatal traits. Genomic recombination via hybridization and subsequent introgression can produce new heritable phenotypes, including traits intermediate or in excess of the range observed in either parent species that may be beneficial for adaptation under changing environmental conditions (Campbell, 2004; Campbell & Wendlandt, 2013; Hamilton & Miller, 2016; Janes & Hamilton, 2017). Thus, identifying genes underlying stomatal variation and tracking their movement across species boundaries may reveal how interspecific gene flow facilitates adaptive trait evolution required for climate adaptation.

One approach to assessing the impact of gene flow into a contact zone and selection against recombination in a hybrid zone is geographic cline analysis, which investigates spatial gradients in traits or allele frequencies where species interbreed (Barton, 1979; Barton & Gale, 1993). Comparing geographic clines across repeated hybrid zones for traits, genome‐wide ancestry, and candidate genes underlying adaptive traits allows an evaluation of how gene flow between species varies across genomic and environmental backgrounds (Stankowski *et al*., 2017; Schield *et al*., 2024). However, as clines may arise from either intrinsic or extrinsic selection, geographic cline analysis alone cannot distinguish the mechanisms facilitating or limiting genetic exchange (Hamilton & Aitken, 2013; Janes & Hamilton, 2017; Capblancq *et al*., 2020). To address this gap, environmental associations may be used to identify the role extrinsic forces may play in adaptive introgression. Integrating geographic cline analyses with environmental associations can help identify traits or genes that move across both environmental and genomic backgrounds and are critical to adaptation under future climates.

*Populus* is an evolutionary and ecological model tree that has become an invaluable system for understanding how interspecific gene flow and natural selection shape trait variation (Taylor, 2002; Buerkle & Lexer, 2008). Stomatal traits in *Populus* exhibit extensive inter‐ and intraspecific variation, largely reflecting adaptation to local climates (Keller *et al*., 2011; Kaluthota *et al*., 2015; Soolanayakanahally *et al*., 2015; Moran *et al*., 2023; Klein *et al*., 2025) Across species the distribution of stomata across leaf surfaces ranges from predominantly hypostomatous (*P. balsamifera*, *P. tremuloides*) to amphistomatous (*P. deltoides*), with intermediate patterns in other species that vary across climatic gradients (Pearce *et al*., 2006; McKown *et al*., 2014, 2019; Kaluthota *et al*., 2015). Within species, as observed previously in *P. angustifolia* and *P. trichocarpa*, more northern genotypes generally tend to have higher adaxial stomatal density than southern genotypes (Kaluthota *et al*., 2015; McKown *et al*., 2019; Blasini *et al*., 2021). Previous genetic studies have identified conserved regulators of stomatal development (e.g. *ERECTA*, *MUTE*, *FAMA*) and natural variation in candidate genes, including *SPEECHLESS*, *GAUT9*, *KCS* clusters, and *WRKY68* associated with density, distribution, and size (Hamanishi *et al*., 2012; Weng *et al*., 2012; McKown *et al*., 2014; Chhetri *et al*., 2019; Liu *et al*., 2021; Fang *et al*., 2023; Li *et al*., 2023; Klein *et al*., 2025). However, much less is known about the extent to which alleles introgress across species boundaries and how these dynamics shape the evolution of stomatal traits. Here, we address this gap by quantifying how genetic variation underlying stomatal traits moves across species boundaries. We specifically focus on the natural hybrid zone between *P. trichocarpa* × *P. balsamifera*, two sister species that have evolved in response to contrasting environmental conditions (Bolte *et al*., 2024). *P. trichocarpa* occupies mild, moist environments of the Pacific Northwest and typically exhibits amphistomaty, with large stomata on both leaf surfaces (McKown *et al*., 2014, 2019; Pointeau & Guy, 2014; Richardson *et al*., 2014). By contrast, *P. balsamifera*, which is adapted to colder, drier boreal regions that experience greater seasonal extremes, generally exhibits hypostomaty, with smaller, denser stomata concentrated on the lower leaf surface (Geraldes *et al*., 2014; Pointeau & Guy, 2014; Richardson *et al*., 2014). Previous work has indicated that the movement of genomic regions from *P. balsamifera* into the genomic background of *P. trichocarpa* has contributed to adaptive trait evolution linked to persistence in colder and drier environments (Suarez‐Gonzalez *et al*., 2018a,b). However, less is known about how genetic variation underlying stomatal traits moves across species boundaries (but see Fetter & Keller, 2023), or how climate may influence that movement.

Here, we identify the genetic variation underlying stomatal traits and compare the broad‐ and fine‐scale roles of geography and climate in shaping the movement of genetic variation across repeated contact zones between *P. trichocarpa* and *P. balsamifera*. Specifically, we ask: (1) How do hybrids vary in stomatal traits relative to *P. trichocarpa* and *P. balsamifera*? (2) To what extent do stomatal traits vary across repeated contact zones, and are these patterns shaped by local climatic gradients? (3) What genes underlie stomatal trait variation, and how does the environment interact to influence the extent and direction of introgression for candidate genes across contact zones? This work identifies key candidate genes related to stomatal trait variation in *Populus* and their relationships with climate gradients, providing a basis to predict whether and how genetic variation underlying adaptive traits may shift across species and environmental gradients under climate change.

## Materials and Methods

### Study approach

#### Sample collection and library preparation

**Fig. 1:**
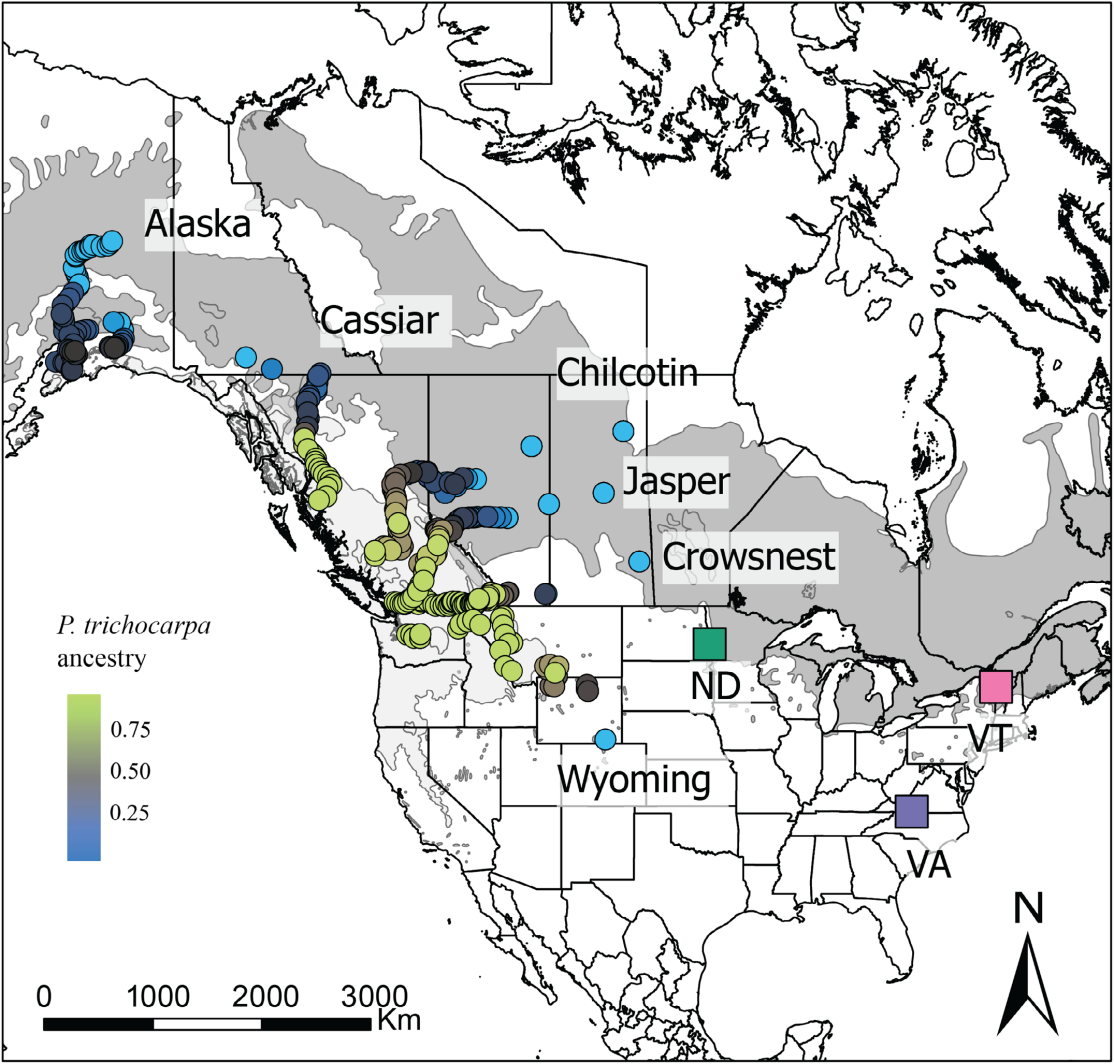
Sampling and common garden design. Genotypes were collected across six east–west contact zones within the natural hybrid zone between *Populus trichocarpa* and *Populus balsamifera*. Light and dark gray represent the distribution range of *P. trichocarpa* and *P. balsamifera*, respectively. Georeferenced sampling locations are color coded by genomic ancestry. Clonally replicated genotypes were planted at the common gardens in Stuart, VA (purple square), Burlington, VT (pink square), and Fargo, ND (green square).

In this study, we analyzed 574 genotypes collected within the natural contact zone between *Populus trichocarpa* (Torr. & Gray) and *Populus balsamifera* (L.) hybrid zone (Fig. 1; Supporting Information Table S1; see Bolte *et al*., 2024 for full details). A minimum of 100 dormant vegetative cuttings were sampled from each of six contact zones spanning a latitudinal gradient to capture environmental variation across latitude. The contact zones are referred to here as Alaska, Cassiar, Chilcotin, Jasper, Crowsnest, and Wyoming. Cuttings were propagated clonally under glasshouse conditions (24°C : 15.5°C, day : night, no supplemental lighting) at the Virginia Tech Reynold's Homestead Forest Resource Research Center (FRRC) in Stuart, VA, USA. Once established, 100 mg of fresh leaf tissue was collected from each genotype for DNA extraction and whole‐genome resequencing. Methods for propagation, DNA extraction, library preparation, sequencing, and quality filtering are fully described in Bolte *et al*., 2024. Genomic libraries were sequenced on an Illumina NovaSeq 6000 using an S4 flow cell in 2 × 150 bp paired‐end format, with 64 samples per lane. Illumina reads for each genotype were aligned to the *P. trichocarpa* reference genome (v.4.0), generating SAM files that were subsequently converted to BAM format using samtools (Li *et al*., 2009). Individual gVCF files were generated using the HaplotypeCaller algorithm in GATK v.3.7 and subsequently combined into a single VCF file using the GenotypeGVCFs function. The initial dataset included *c*. 82 million variants and was filtered based on mapping quality (MQ < 40.00), strand bias (FS > 40.000, SOR > 3.0), mapping quality rank sum (MQRankSum < −12.500), read position bias (ReadPosRankSum < −8.000), and depth of coverage (QD < 2.0). INDELs and SNPs with more than two alternate alleles were excluded and then filtered to remove SNPs with a minor allele frequency below 5% and > 10% missing data, resulting in a final dataset of 7361 750 biallelic SNPs. Additionally, 30 individuals were excluded based on ancestry estimates from Bolte *et al*. (2024) as their genomic composition did not correspond to either parental species, resulting in 544 genotypes and 7167 726 biallelic SNPs for the downstream analysis. The final sample sizes per contact zone were Alaska (*n* = 80), Cassiar (*n* = 76), Chilcotin (*n* = 120), Jasper (*n* = 97), Crowsnest (*n* = 94), and Wyoming (*n* = 77).

#### Common garden design

In March 2020, the 574 clonally propagated rooted *Populus* cuttings were planted across three common garden environments (Fig. 1). The common gardens, located at the FRRC in Stuart, VA (36°37′N and 80°09′W, elevation 359 m), Burlington, VT (44°26′N and 73°11′W, elevation 130 m), and Fargo, ND (46°53′N and 96°48′W, elevation 272 m) were established spanning both latitudinal and longitudinal gradients in temperature and precipitation. At each site, genotypes were planted in a randomized complete block design with three replicates per genotype, resulting in a total of 1722 individuals per site.

#### Quantifying stomatal traits in replicated common gardens

During the summer of 2022, stomatal traits and associated measures were assessed for each genotype across three common garden experiments (see Table 1 for list). For each genotype, the first fully expanded neoformed leaf on the dominant shoot was used to assess stomatal conductance, size, density, and distribution on both the adaxial (upper) and abaxial (lower) leaf surfaces. This leaf was selected to minimize variation due to leaf age and environmental effects between samples (Fetter *et al*., 2021). Stomatal conductance (*g*_sw_, mmol m^−2^ s^−1^) was measured using a LI‐600 porometer in three nonoverlapping areas of each fully expanded leaf without detaching the leaf from the plant. Individual stomatal conductance values represent the average of these three measurements. To limit variability in stomatal conductance, which is an environmentally responsive trait, we confined sampling to mornings (08:00 h–11:00 h) with no cloud cover or rainfall.

**Table 1:** Summary of *Populus* stomatal traits and associated measures assessed across the common gardens, including trait names, abbreviations, units, and symbology. Trait differences among parental species and hybrids are based on results from our study indicating whether hybrids showed intermediate values or resembled one parent. ns, no significant differences among groups based on *post‐hoc* comparisons. Stomata drawings were created in BioRender. Zavala Paez, M. (2025) https://BioRender.com/f306wfh

Trait	Units	Symbology	*P. balsamifera*	Hybrids	*P. trichocarpa*
Adaxial guard cell length (*G*_U_)	μm	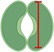	ns	ns	ns
Abaxial guard cell length (*G*_L_)	μm		Greater	*P. balsamifera*‐like	Smaller
Adaxial pore length (*P*_U_)	μm	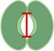	ns	ns	ns
Abaxial pore length (*P*_L_)	μm		Greater	*P. balsamifera*‐like	Smaller
Adaxial stomatal density (*D*_U_)	mm^2^	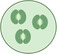	None or less dense	*P. trichocarpa*‐like	Denser
Abaxial stomatal density (*D*_L_)	mm^2^		Less dense	*P. balsamifera*‐like	Denser
Total stomatal density (*D*_T_)	mm^2^	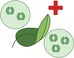	Less dense	*P. balsamifera*‐like	Denser
Stomatal ratio (SR)	None	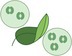	More hypostomatous	*P. trichocarpa*‐like	Higher degree of amphistomy
Adaxial stomatal occurrence (*O*_U_)	None	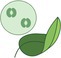	Lower	*P. trichocarpa*‐like	Higher
Stomatal conductance (*g*_sw_)	mol m^−2^ s^−1^	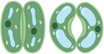	Higher	Intermediate values	Lower
Intrinsic water‐use efficiency (δ^13^C)	‰	High/Low WUE	Lower	*P. balsamifera*‐like	Higher

Following stomatal conductance measurements, leaves were collected to assess stomatal density, size, and distribution. A layer of Newskin liquid bandage was applied to the upper and lower leaf surfaces to make two stomatal impressions to capture variation across leaf surfaces. Stomatal impressions from one leaf per individual were mounted on slides without a cover slip, and images were captured using an Olympus BX‐53 microscope equipped with an Olympus DP23 digital camera. Micrographs were standardized to a 0.37 × 0.25 mm grid for analysis. Stomatal size traits were measured and analyzed independently for each leaf surface using imagej (Abràmoff *et al*., 2004). These included adaxial (*G*_U_, μm) and abaxial (*G*_L_, μm) guard cell length, as well as adaxial (*P*_U_, μm) and abaxial (*P*_L_, μm) pore length. Stomatal measurements were obtained by overlaying four equally spaced lines across each micrograph, selecting five stomata per image. Individual values represent the average of five measurements per leaf surface. Stomatal density was estimated as the number of stomata per unit leaf area for the adaxial (*D*_U_, mm^−2^) and abaxial (*D*_L_, mm^−2^) leaf surfaces using automated counts in LeafNet (Li *et al*., 2022). To validate automated density measures, micrographs from 100 random genotypes were manually counted and measured. A strong correlation was observed between automated and manual counts (*ρ* = 0.95, *P* < 0.05, Fig. S1); therefore, automated values generated by LeafNet were used for all genotypes. Total stomatal density (*D*_T_) was also calculated as the sum of adaxial and abaxial stomatal density, representing the overall number of stomata per unit leaf area. To estimate the degree of amphistomy, the stomatal ratio (SR) was calculated as the ratio of adaxial stomatal density divided by the total stomatal density. Stomatal ratio values range from 0 to 1, with 0 indicating stomata are present only on the abaxial surface, 0.5 indicating equal density on both surfaces, and 1 indicating stomata are exclusive to the adaxial surface (Fetter *et al*., 2021). To capture variation in adaxial stomatal occurrence (O_U_) among genotypes we assessed the presence (1) or absence (0) of adaxial stomata.

In addition to stomatal trait variation, intrinsic water‐use efficiency (δ^13^C, ‰) was assessed using the second fully expanded leaf. δ^13^C reflects the balance between CO₂ supply through stomata and CO_2_ assimilation during photosynthesis (Farquhar *et al*., 1982; Volk *et al*., 2022). Leaves were collected and dried at 60°C until a constant mass was reached. Dried leaf tissue was then homogenized into a fine powder using a TissueLyser II (Qiagen, Hilden, Germany). Approximately 2–3 mg of the homogenized tissue was placed into a tin capsule (Costech, Valencia, CA, USA) for analysis. Carbon isotope composition (δ^13^C) was measured at the Central Appalachians Stable Isotope Facility (CASIF), Appalachian Laboratory (Frostburg, MD, USA).

### Comparing variation in stomatal traits among hybrid genotypes relative to parental species

To test whether hybrids exhibit intermediate, transgressive, or parental‐like stomatal traits, we first estimated the genetic values of each genotype for each trait by calculating best linear unbiased predictors (BLUPs) using R 4.3.1 (R Core Team, 2021). BLUPs were estimated for each trait for each genotype across the three common garden experiments using the following linear mixed model.

$$y_{\text{ijkl}}\sim \mu +E_{i}+B_{i\left(j\right)}+G_{k}+\varepsilon_{\text{ijkl}}$$

where $y_{\text{ijkl}}$ is the observed trait value for clonal replicate *l*, $E_{i}$ is the fixed effect of the common garden experiment, $B_{i\left(j\right)}$ is the random effect of block *j* nested within garden *i*, $G_{k}$ is the random effect of genotype, and $\varepsilon_{\text{ijkl}}$ is the residual term. We then extracted the genotype random effects ($G_{k}$), which represents each genotype's estimated genetic contribution to the trait after accounting for garden and block effects for use in downstream analyses.

Individuals were classified into three genotypic classes based on admixture proportions from Mead *et al*. (2025, see Methods S1 for details) to evaluate species‐specific and hybrid differences in stomatal traits. Using ancestry estimates (*K* = 2), genotypes with *Q* > 0.98 were assigned to *P. trichocarpa* (*n* = 132) and those with *Q* < 0.02 to *P. balsamifera* (*n* = 91). Remaining genotypes (*n* = 323) were categorized as hybrids. To test for differences in stomatal traits among *P. trichocarpa*, *P. balsamifera*, and their hybrids, we first assessed homogeneity of variance using Levene's test in R 4.3.1 (RStudio Team, 2024). For traits that met the assumptions of homogeneity, we used one‐way ANOVA followed by Tukey's HSD for *post hoc* comparisons. For traits that violated this assumption, we applied the nonparametric Kruskal–Wallis test with Dunn's *post hoc* comparisons. Four out of eleven stomatal traits, including stomatal conductance, stomatal ratio, adaxial stomatal density, and occurrence, did not exhibit homogeneity of variance across groups and were therefore analyzed using the nonparametric tests.

### Admixture mapping to identify the genetic basis of stomatal variation

To identify the genetic basis of stomatal trait variation and water‐use efficiency, we performed admixture mapping using local ancestry inferred with Loter (Dias‐Alves *et al*., 2018) and phenotype–genotype association conducted with GEMMA (Zhou & Stephens, 2014). Local ancestry inference relies on phased haplotypes from reference parental genotypes to infer the ancestral origin of specific genomic regions in admixed genotypes (Dias‐Alves *et al*., 2018). Before local ancestry inference, the VCF file was phased and missing data imputed using BEAGLE (Browning *et al*., 2018, 2021). Parental reference genotypes for local ancestry inference were the same as those used for trait comparisons as defined above. These phased reference haplotypes were used to infer local ancestry across the genomes of 323 admixed individuals.

To associate genomic variation with trait variation, we used Univariate Linear Mixed Models implemented in GEMMA v.0.94.1 (Zhou & Stephens, 2014). A relatedness matrix (estimated with the ‐gk 1 option) was included as a covariate to account for relatedness among individuals. Global ancestry, estimated as the average local ancestry across all loci, was also included as a covariate to account for population structure (Fetter & Keller, 2023). To account for multiple testing, the significance threshold was adjusted using the admixture burden (0.05/5055), which estimates the number of independently recombining chromosomal segments within a genotype. Admixture burden was estimated following the method described by Shriner *et al*. (2011). Specifically, an autoregressive (AR) model was fitted to the local ancestry sequence of each genotype, and the spectral density of local ancestry values at frequency zero was estimated using the spectrum0.ar function from the coda package in R 4.3.1. The number of independent ancestry blocks per individual was then summed and averaged across all individuals to estimate the mean effective number of independent tests in our dataset (Shriner *et al*., 2011). This value was used to adjust the significance threshold for admixture mapping. Manhattan plots were generated using the qqman package in R to visualize admixture mapping results, with significance assessed based on the admixture burden threshold (Turner, 2018). QQ plots were visualized to evaluate whether population structure and relatedness were adequately controlled in the association analyses.

To identify candidate genes associated with stomatal traits, we mapped loci that passed the admixture burden threshold to the *P. trichocarpa* v.4.1 gene annotation. To account for upstream regulatory regions, gene boundaries were extended by 2 kb upstream (positive strand) or downstream (negative strand). We performed manual blast searches on TAIR (www.arabidopsis.org) to identify orthologous genes in *Arabidopsis thaliana* and selected the best hits based on sequence similarity. When multiple genes were located within the same window, all were initially retained as candidates. Each gene was then evaluated for its potential role in stomatal development and function. Candidate gene‐specific ancestry values were extracted from the ancestry matrix using gene positions from the *P. trichocarpa* v.4.1 reference genome, including a ± 500 bp flanking region. For each admixed individual, locus‐specific ancestry at each gene was classified as homozygous *P. trichocarpa* (1), homozygous *P. balsamifera* (0), or heterozygous (0.5).

### Geographic clines to identify barriers to gene flow for stomatal traits, candidate genes, and genome‐wide ancestry

To quantify the extent and direction of introgression for stomatal traits and associated candidate genes, we fitted geographic clines separately for each of the six contact zones. Geographic distances from the coast for each genotype within each contact zone were calculated using the Haversine formula and scaled between 0 and 1 to allow comparisons across contact zones (see Methods S1). Geographic cline parameters, including center and width (1/maximum slope, Derryberry *et al*., 2014), were estimated using genotype‐specific BLUP values for traits and ancestry genotypes for candidate genes using HZAR (Derryberry *et al*., 2014).

For each trait, six different cline models were fit for each contact zone using different tails (both, left, right, mirrored, none, null). Each model was run with a burn‐in of 100 000 iterations followed by 500 000 Monte Carlo iterations to estimate posterior distributions of cline parameters. The best‐supported model for each trait in each contact zone was identified using the corrected Akaike information criterion (AICc), selecting the model with the lowest AICc score. Cline parameter estimates based on the best‐fit model were compared across contact zones.

For each candidate gene, 10 different cline models using different combinations of scaling (free or fixed) and tails (both, left, right, mirrored, or none), in addition to the null model (no cline) were fit for each geographic contact zone. The inclusion of scaling and tail combinations allowed us to capture the full range of potential variation in ancestry genotypes across the contact zones. As with the trait clines, each gene cline model was run with 100 000 burn‐in iterations and 500 000 Monte Carlo iterations. AICc scores were used to select the best‐fitting model for each candidate gene and contact zone, and cline parameter estimates from these models were compared across contact zones. We compared fine‐scale introgression of candidate genes to genome‐wide ancestry by estimating genome‐wide cline parameters for each contact zone and assessing whether candidate gene centers or widths fell within the 95% CI of the genome‐wide ancestry cline.

### Modeling the influence of climate on stomatal traits, candidate genes, and genome‐wide ancestry

To complement geographic cline analyses, which identify barriers to gene flow across space, we modeled climatic clines to test whether climate predicts variation in stomatal traits, candidate genes, and genome‐wide ancestry within each contact zone. Twenty‐five annual climate normals (1961–1990) were obtained from ClimateNA (Wang *et al*., 2016) using the latitude, longitude, and elevation of each genotype origin. Climate normals from 1961 to 1990 were used as they represent the historical climatic conditions that shaped genetic variation in these genotypes. Due to correlations among climate variables, we conducted a principal component analysis (PCA) using the prcomp function in R 4.3.1 to reduce multicollinearity and summarize climatic variation across genotypes based on the climate of origin. PC1 explained 55% of climate variation and captured temperature‐related gradients, with high loadings for mean annual temperature and degree‐days above 5°C and 18°C. PC2 explained 26% of climate variation and captured moisture‐related gradients, with high loadings for mean annual precipitation, climate moisture index, and relative humidity (Table S1; Fig. S2). Given that PC1 and PC2 encompassed the major axes of climate variation, subsequent analyses focused on these two PC axes.

For each trait and contact zone, linear regressions were fit to quantify the relationship between climate and trait variation. Genotype‐specific BLUP values were used as the response variable and the first two climate principal components (PC1 and PC2) as predictors within the lm function in R 4.3.1 (R Core Team, 2021) following the equation:

$$y_{k}\sim \mu +E1_{k}+E2_{k}+\varepsilon_{k}$$

where $y_{k}$ is the BLUP‐estimated trait value for genotype *k*, *μ* is the intercept, and E1 and E2 represent the scores for PC1 (temperature‐related gradient) and PC2 (moisture‐related gradient), respectively, and $\varepsilon_{k}$ is the residual error term. An interaction term between PC1 and PC2 was included in initial models but was not significant for the majority of trait–contact zone combinations. To assess whether the direction and strength of climate–trait relationships varied across transects, slopes were compared across contact zones.

For each candidate gene, we tested whether variation in gene ancestry was associated with climatic gradients using logistic regression models implemented within the glm() function in R 4.3.1. We fit a single model for each gene in each contact zone, including both PC1 and PC2 as well as their interaction (PC1 × PC2) as predictors following the equation:

$$\text{logit}\left(P\left(y_{k}\right)\right)\sim \mu +E1_{k}+E2_{k}+E1_{k}*E2_{k}+\varepsilon_{k}$$

where, $y_{k}$ is the candidate gene local ancestry value for genotype *k*, μ is the intercept, and E1 and E2 represent the scores for PC1 (temperature‐related gradient) and PC2 (moisture‐related gradient), respectively. $E1_{k}*E2_{k}$ represents the interaction term between PC1 and PC2, and $\varepsilon_{k}$ is the residual error. To compare climatic associations between candidate genes and the genome‐wide average, the same model was fit using genome‐wide ancestry as the response variable. Slope values from candidate gene models were compared to those from the genome‐wide models within each contact zone to evaluate differences in the strength and direction of climate–ancestry relationships. Slopes were also compared across contact zones for each candidate gene to evaluate whether climate–gene ancestry relationships vary.

## Results

### Species‐specific and hybrid variation in stomatal traits between *P. balsamifera* and *P. trichocarpa*

**Fig. 2:**
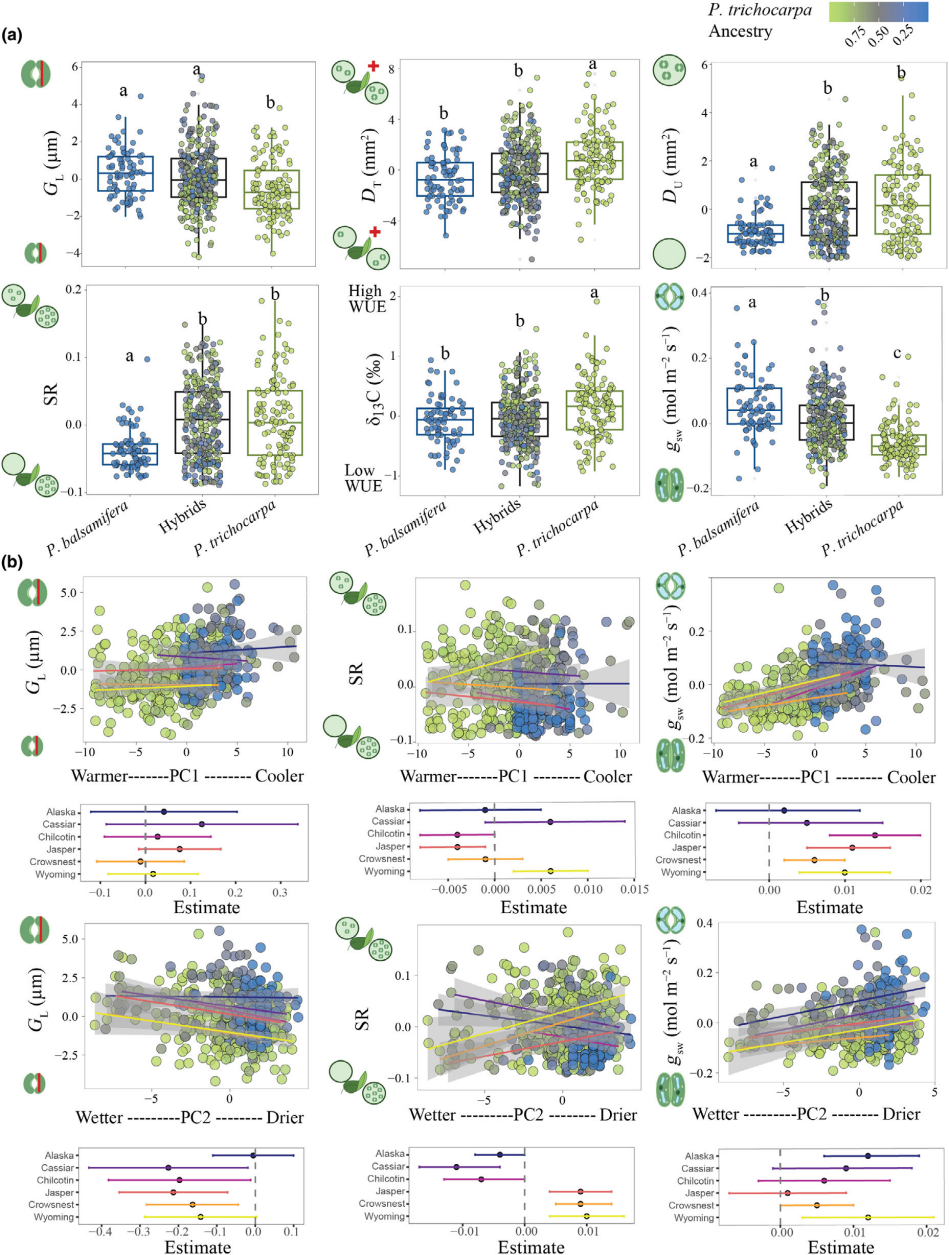
Variation in stomatal traits across genotypic classes and climate gradients. (a) Boxplots showing best linear unbiased predictors (BLUPs) for abaxial guard cell length (*G*_L_), total stomatal density (*D*_T_), adaxial stomatal density (*D*_U_), stomatal ratio (SR), intrinsic water‐use efficiency (δ^13^C), and stomatal conductance (*g*_sw_) for *Populus balsamifera*, hybrid, and *Populus trichocarpa* genotypes. Each point represents a genotype, and colors indicate the proportion of *P. trichocarpa* genomic ancestry. Letters denote statistically significant differences among groups based on *post‐hoc* comparisons (*P* < 0.05). The central line represents the median, the box bounds correspond to the interquartile range (IQR; 25^th^–75^th^ percentiles), whiskers extend to 1.5 × IQR, and points beyond whiskers represent outliers. (b) Relationship between genotype BLUPs values for *G*_L_, SR, *g*_sw_, and the first two principal components (PC) of climate at genotypes' origin. PC2 reflects a gradient from wetter to drier environments (increasing values = drier), while PC1 reflects a temperature gradient (increasing values = warmer). Shaded areas indicate 95% confidence intervals (CIs) for the fitted linear regression. Lower panels show trait‐transect slopes with line bars representing the ±95% CIs. Stomata drawings were created in BioRender. Zavala Paez, M. (2025) https://BioRender.com/f306wfh.

Parental genotypes of *P. trichocarpa* and *P. balsamifera* differed significantly in several stomatal traits, with hybrids exhibiting intermediate values or resembling one of the parental species (Fig. 2a; Table 1). On average, *P. trichocarpa* exhibited reduced abaxial guard cell length compared to *P. balsamifera* (*P* < 0.05; Fig. 2a; Table S2), while adaxial guard cell length did not differ significantly between parental species (Fig. S3). In hybrids, abaxial guard cell length was similar to *P. balsamifera* (*P* > 0.05; Fig. 2a); however, hybrids exhibited a broader range than either parental species. Overall, adaxial guard cell (*F*_2,496_ = 9.36, *P* > 0.05) and adaxial pore length (*F*_2,386_ = 2.91, *P* > 0.05; Fig. S3) were not significantly different between parental and hybrid genotypes. By contrast, abaxial pore length differed significantly among genotype classes (*F*_2,496_ = 4.39, *P* < 0.05, Fig. S3); *post hoc* comparisons showed that hybrid genotypes had significantly greater abaxial pore length than *P. trichocarpa* (*P* < 0.05) but did not differ from *P. balsamifera* (*P* > 0.05). Hybrids also exhibited a greater range of abaxial pore length values relative to parental species.

*Populus balsamifera* had significantly lower total stomatal density than *P. trichocarpa* (*P* < 0.05; Fig. 2a). Hybrid genotypes did not differ significantly from *P. balsamifera* (*P* > 0.05), but their total stomatal density was significantly lower than *P. trichocarpa* (*P* < 0.05), and their range of phenotypic values exceeded both parental species. Abaxial stomatal density did not differ significantly between the parental species (*P* < 0.05), although *P. trichocarpa* tended to exhibit higher abaxial stomatal density than *P. balsamifera* (Fig. S3). Hybrid genotypes had significantly lower abaxial stomatal density than *P. trichocarpa* (*P* < 0.05) but did not differ from *P. balsamifera* (*P* > 0.05). Compared to both parental species, the range of hybrid abaxial stomatal density values was greater. *P. trichocarpa* exhibited significantly greater adaxial stomatal occurrence and density than *P. balsamifera* (*P* < 0.05; Fig. 2a). Hybrid genotypes did not differ significantly from *P. trichocarpa*, with greater adaxial stomatal occurrence and a comparable range of trait values. *P. trichocarpa* exhibited a more even distribution of stomata across leaf surfaces, reflected in a significantly higher stomatal ratio compared to *P. balsamifera*, which had most stomata restricted to the lower surface (*P* < 0.05, Fig. 2a). Hybrids resembled *P. trichocarpa*, with a higher stomatal ratio (*P* > 0.05) and a similar range of values. *P. trichocarpa* showed significantly lower stomatal conductance and higher water‐use efficiency (δ^13^C) than *P. balsamifera* (*P* < 0.05; Fig. 2a). In hybrids, stomatal conductance was intermediate between parents but exhibited a broader range of trait values. By contrast, water‐use efficiency values for hybrids more closely resembled *P. balsamifera* (*P* > 0.05), and the range did not exceed that observed in both parentals.

### Contact zone‐specific climatic gradients influenced stomatal traits despite high interspecific gene flow

Geographic cline analyses showed no clinal variation across geographic distance for stomatal traits (Figs S4–S5), yet these traits were consistently associated with climatic gradients of origin, particularly precipitation (PC2) across the six latitudinal contact zones (Fig. 2b; Table S3). Opposing trait–climate relationships were observed between northern and southern contact zones for adaxial stomatal density, occurrence, and stomatal ratio. Drier environmental origins (PC2) were associated with significant decreases in adaxial stomatal density, occurrence, and stomatal ratio in the northern contact zones of Alaska, Cassiar, and Chilcotin, but significantly increased along PC2 in the southern contact zones of Jasper, Crowsnest, and Wyoming (Fig. 2b; Table S3, *P* < 0.05). Genotypes from warmer environments (PC1) were associated with increased adaxial stomatal density and occurrence across the Jasper and Chilcotin contact zones, but lower density and occurrence in the Wyoming contact zone (*P* < 0.05), while no significant associations were observed in the remaining contact zones (Fig. S6). Neither precipitation nor temperature gradients of origin were significantly associated with abaxial or total stomatal density across most contact zones (*P* > 0.05, Fig. S6), except for Alaska and Jasper, where warmer and drier origins were associated with increased total stomatal density (*P* < 0.05).

Genotypes from drier origins (PC2) were associated with reduced abaxial guard cell and pore length across most contact zones (*P* < 0.05), except for Alaska and Wyoming, where similar but not significant associations were observed (*P* > 0.05, Fig. 2b). In contrast to the abaxial surface, adaxial guard cell and pore length did not vary significantly with precipitation gradients from site of origin (*P* > 0.05, Fig. S6) suggesting that stomatal size on the upper surface is influenced less by precipitation. Temperature gradients (PC1) were not significantly associated with guard cell length or pore length on either the adaxial or abaxial surface (*P* > 0.05, Fig. S6).

In contrast to stomatal density and size traits, stomatal conductance was positively associated with temperature (PC1) and precipitation (PC2) gradients of origin across multiple contact zones (Fig. 2b; Table S3). Genotypes from cooler environments exhibited significantly greater stomatal conductance (*P* < 0.05) with the exception of Alaska and Cassiar. However, in Alaska, genotypes from drier environments had higher stomatal conductance (*P* < 0.05). Although no significant associations were detected between climatic gradients and water‐use efficiency (δ^13^C) in most contact zones, warmer genotype origins tended to be associated with reduced water‐use efficiency (Fig. S6). Drier genotype origins were negatively associated with water‐use efficiency in Cassiar and Wyoming (*P* < 0.05), but not in other contact zones (Fig. S6; Table S3), suggesting a trend toward lower water‐use efficiency in drier environments for these contact zones.

### Admixture mapping reveals common and trait‐specific candidate genes

**Fig. 3:**
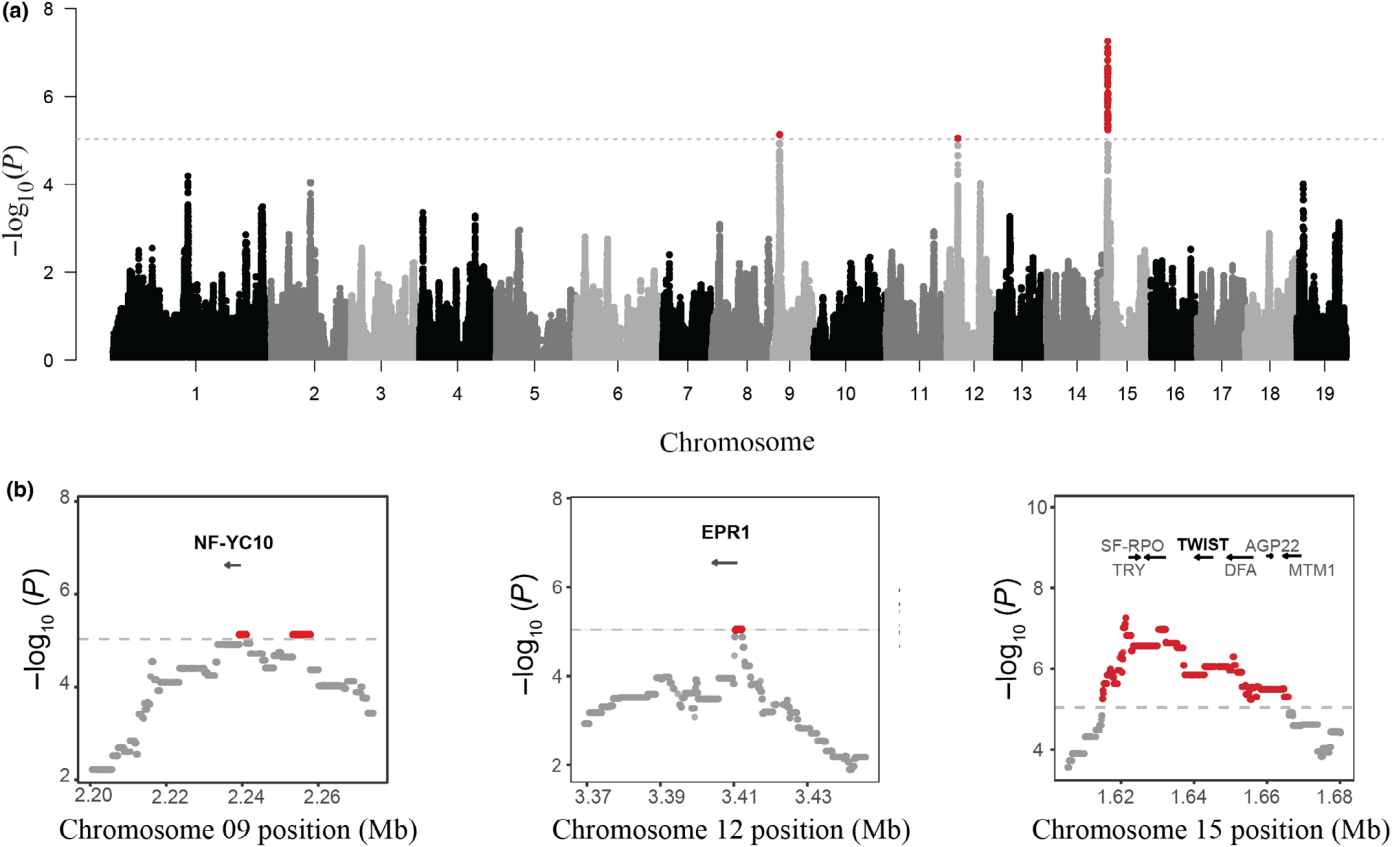
Genome‐wide associations identified for candidate genes underlying stomatal ratio variation. (a) Manhattan plot shows results from genome‐wide association by admixture mapping for stomatal ratio in *Populus*. The black dashed line indicates the genome‐wide significance threshold (−log_10_ (*P*) ≈ 4.73), corrected for admixture burden (0.05/5055). (b) Zoomed‐in views of significant associations with stomatal ratio on chromosomes 9, 12, and 15. Significant SNPs are highlighted in red. Candidate genes and their orientations are indicated by arrows.

Admixture mapping identified multiple ancestry blocks significantly associated with stomatal trait variation in hybrid genotypes (Table S4), revealing both trait‐specific and shared regions of genomic association. The strongest associations were found for stomatal ratio, adaxial stomatal occurrence, and density, which were all associated with the same genomic region on chromosome 15 spanning *c*. 66.5 kb. Multiple loci within this region exceeded the admixture burden‐corrected significance threshold (Figs 3a, S7). The region contained several candidate genes (Fig. 3b), including *TWIST* (Potri.015G022300, a homologue of *SPEECHLESS* in *Arabidopsis*), *TRY* (Potri.015G022000), *AGP22* (Potri.015G022600), *SF‐RPO* (RNA polymerase sigma factor sigE, Potri.015G022100), *DFA* (*dihydrofolate synthetase*, Potri.015G022400), and *MTM1* (Potri.015G022700). In addition, a region on chromosome 12 associated with both adaxial stomata density and stomatal ratio contained *EPR1*(Potri.012G038300; Fig. 3b). A third region on chromosome 9, containing *NF‐YC10* (Potri.009G012300), was associated with stomatal ratio (Fig. 3b). Although no other traits passed the genome‐wide significance threshold, we discuss suggestive associations for several additional traits in Notes S1 (Figs S8–S11).

### Contact zone‐specific geographic clines suggest barriers to gene flow at candidate loci for stomatal traits

**Fig. 4:**
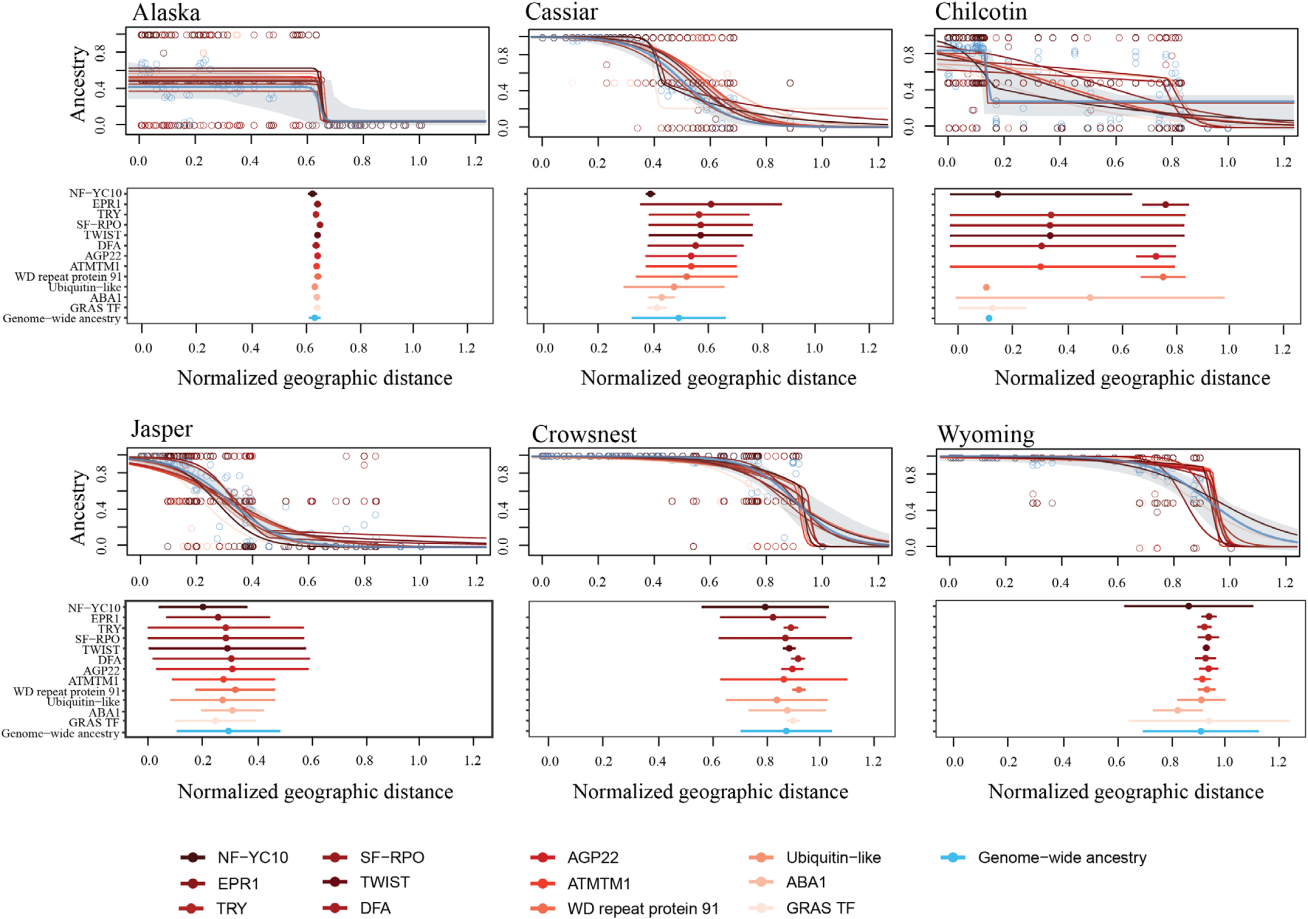
Geographic clines for candidate genes associated with stomatal trait variation and genome‐wide ancestry across six contact zones in the *Populus balsamifera × P. trichocarpa* hybrid zone. Best‐fit cline models estimated using HZAR illustrate the relationship between scaled geographic distance and ancestry (proportion of *P. trichocarpa* ancestry). Each colored line and dot represents a candidate gene, with cline shape determined by the model that best fits the observed ancestry frequency transition across the landscape. The gray shaded area represents the 95% confidence interval for the genome‐wide ancestry best‐fit model. The lower panel provides a summary of cline parameters for each candidate gene. Points represent the estimated cline center (geographic midpoint of ancestry frequency change), and horizontal bars denote cline width (the spatial scale over which the transition occurs).

Geographic cline analyses revealed variation in cline centers and widths for candidate genes across the six *Populus* contact zones (Fig. 4; Table S5). In the Alaska contact zone, all stomatal candidate genes exhibited steep clines with centers and widths within the confidence interval (CI) of the genome‐wide ancestry cline (*w* = 0.04, 95% CI: 0.00–0.58; *c* = 0.64, 95% CI: 0.48–0.690). Cline centers for these candidate genes were clustered towards the geographic distribution of *P. balsamifera* (*c* ≈ 0.65), and cline widths were narrow across candidate genes (*w* < 0.03). By contrast, candidate genes within the Jasper cline were broader (*w* = 0.23–0.58), with centers ranging from *c* = 0.23 to 0.35. For this contact zone, cline parameters fell within the 95% CIs of the genome‐wide ancestry cline interval.

In the Cassiar, Chilcotin, Crowsnest, and Wyoming contact zones, several candidate genes exhibited steeper clines when compared to the genome‐wide background (Fig. 4; Table S5). In Cassiar, the cline for genome‐wide ancestry was relatively broad (*w* = 0.34, 95% CI: 0.23–0.56) and centered around *c* = 0.51 (95% CI: 0.45–0.57). This contrasted with several candidate genes associated with adaxial stomatal density, conductance, and guard cell size, such as *NF‐YC10*, *ABA1*, and *GRAS transcription factor*, which exhibited steeper clines (*w* = 0.03–0.09) with centers (*c* = 0.40–0.44) consistently displaced toward the *P. trichocarpa* range. In Chilcotin, the genome‐wide ancestry cline was extremely narrow (*w* = 0.02, 95% CI: 0.00–0.09), but clines at candidate genes varied widely, reflecting candidate gene‐specific patterns of introgression. Genes associated with adaxial stomatal density, *TRY*, *TWIST*, and *DFA*, exhibited wide clines (*w* = 1.00), with centers displaced toward the *P. trichocarpa* range (*c* = 0.38, 0.37, and 0.34, respectively). By contrast, *AGP22* (adaxial stomatal density) and *WD repeat protein 91* (total stomatal density) showed narrower clines (*w* = 0.14 and 0.16) with centers at *c* = 0.77 and 0.80, respectively. In Crowsnest, candidate genes associated with adaxial stomatal density including *TRY* (*w* = 0.05), *TWIST* (*w* = 0.04), *DFA* (*w* = 0.05), and *AGP22* (*w* = 0.08) exhibited narrower clines than the genome‐wide average (*w* = 0.33, 95% CI: 0.20–0.66), while cline centers (*c* = 0.91–0.95) were within the genome‐wide CI center (*c* = 0.90, 95% CI: 83–0.99). In Wyoming, the genome‐wide ancestry cline was relatively broad (*w* = 0.42, 95% CI: 0.23–0.89), with GRAS transcription factor (*w* = 0.59) and NF‐YC10 (*w* = 0.47) cline widths within the 95% CI of the genome‐wide ancestry. However, despite those patterns, the majority of candidate genes had steeper transitions with cline widths ranging from 0.02 to 0.18. Cline centers were largely consistent across candidate genes, ranging from 0.84 to 0.95 and closely aligned with the genome‐wide center (*c* = 0.92, 95% CI: 0.84–0.99).

### Climatic associations suggest extrinsic selection shapes local ancestry at genes associated with stomatal traits

**Fig. 5:**
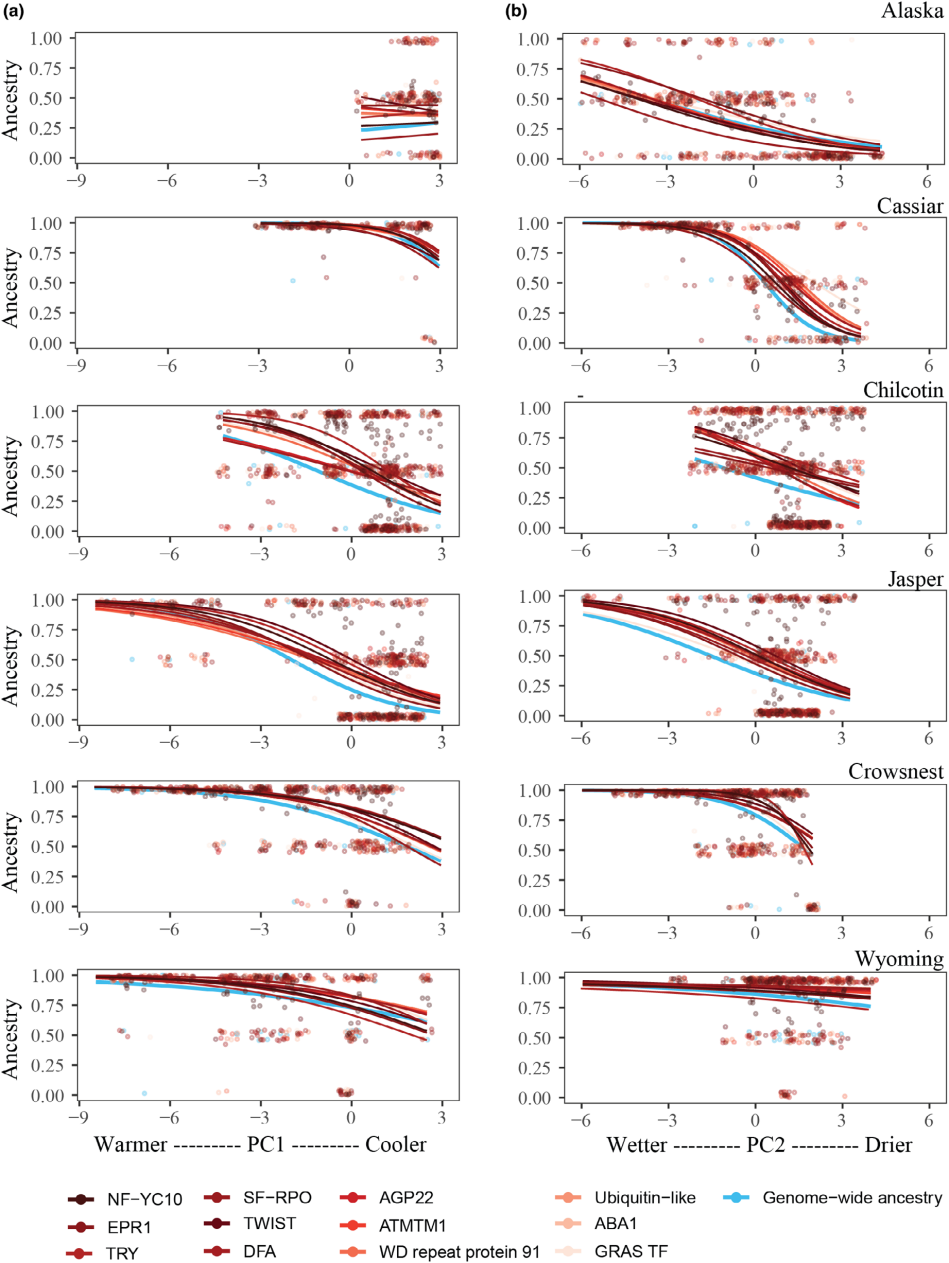
Relationship between climate and ancestry at stomatal trait candidate genes across six *Populus* contact zones. (a) Ancestry as a function of PC1, representing a gradient from warmer (low values) to cooler (high values) environments. (b) Ancestry as a function of PC2, representing a gradient from wetter (low values) to drier (high values) environments. Each point represents an individual genotype, colored by gene. Lines represent fitted values from logistic models, fitted separately for each gene within each contact zone, with ancestry coded as 0 (*P. balsamifera* homozygotes), 0.5 (heterozygotes), or 1 (*P. trichocarpa* homozygotes).

Precipitation gradients associated with the climate of origin had the strongest influence on local ancestry at stomatal trait candidate genes across contact zones (Fig. 5, Table S6). Precipitation gradients (PC2) were associated with reduced *P. trichocarpa* local ancestry (*P* < 0.01) at most candidate genes across Alaska, Cassiar, Chilcotin, Jasper, and Crowsnest contact zones (Figs S12). The probability of *P. trichocarpa* ancestry declined in genotypes originating from drier environments (slopes: −0.25 to −4.2), highlighting strong climate‐mediated selection favoring *P. balsamifera* alleles where water availability was reduced. In Cassiar, genes associated with adaxial stomatal density and conductance, such as *ABA1*, *SF‐RPO*, *TRY*, and *TWIST*, exhibited the steepest declines in *P. trichocarpa* ancestry along precipitation gradients (slopes = −2.8 to −4.23).

Temperature gradients (PC1) based on genotype origin were significantly negatively associated with local ancestry for candidate genes across several contact zones, although these effects were generally weaker and less consistent than those driven by precipitation (PC2, Figs S12). In the Chilcotin, Jasper, and Crowsnest contact zones, colder environmental origins were consistently associated with reduced presence of *P. trichocarpa* alleles at candidate genes, with slopes ranging from −0.27 to −1.29. In Crowsnest, genes associated with adaxial stomatal density and conductance, such as *ABA1*, *SF‐RPO*, *TRY*, and *TWIST*, exhibited the steepest declines in *P. trichocarpa* ancestry along temperature gradients (slopes = −0.66 to −1.29). In Alaska and Cassiar, temperature gradients had limited influence on local ancestry, with only a few genes (*NF‐YC10*, *AGP22*, *ATMTM1*, *ABA1*) showing significant but moderate declines in *P. trichocarpa* ancestry in cooler environments (slopes: −0.33 to −1.06).

Local ancestry at stomatal trait candidate genes was influenced by the interaction between temperature and precipitation gradients from the climate of origin in the Cassiar and Chilcotin contact zone (Fig. S12; Table S6). In Cassiar, genes involved in stomatal conductance and patterning, such as *ABA1*, *AGP22*, and *TWIST*, exhibited significant negative PC1 × PC2 interactions (*P* < 0.05), indicating selection for *P. balsamifera* ancestry under cold and dry conditions. In Chilcotin, similar interactions at multiple genes, including *NF‐YC10*, *EPR1*, and *GRAS TF*, were found. Interestingly, when individual candidate genes were compared with genome‐wide ancestry, consistent declines in *P. trichocarpa* allele frequencies were observed under colder and drier conditions across the six contact zones (Fig. 5).

## Discussion

As climate change accelerates, understanding how gene flow and selection interact to shape the evolution of adaptive traits will be critical to guiding species management. In this study, we investigated how interspecific gene flow and climate‐mediated selection influence the evolution of stomatal traits that are crucial to plant growth, survival, and water‐use efficiency across six natural contact zones between *P. trichocarpa* and *P. balsamifera*. Hybrid genotypes exhibited intermediate or parent‐like phenotypes for key stomatal traits, but there was also substantial genetic variation mediated via hybridization. At a trait scale, geographic cline analyses indicated high genetic exchange; however, analysis of candidate genes underlying stomatal traits showed steep clines aligned with climatic gradients. Such gene‐specific patterns indicate that extrinsic selection plays an important role in influencing the movement of specific alleles across environmental gradients, even in the presence of species barriers associated with adaptive trait differences. Together, our results provide new insight into how extrinsic selection impacts the movement of genetic variants into novel genomic and environmental backgrounds that may be critical to adaptive trait evolution.

### Species‐specific differences in stomatal traits influence hybrids

Stomatal trait differences between *P. balsamifera* and *P. trichocarpa* reflect evolutionary responses to their climate of origin. In this study, traits associated with water conservation, such as reduced stomatal conductance, a high density of small stomata, and increased water‐use efficiency, were observed in *P. trichocarpa* compared to *P. balsamifera*. This contrasts with earlier findings that associated *P. balsamifera* with more water conservation strategies (Pointeau & Guy, 2014). One possibility is that the common garden locations represent novel extremes for *P. trichocarpa* relative to *P. balsamifera*. Thus, *P. trichocarpa* may have experienced lower water potentials in our controlled, comparative environments, leading to stomatal closure to reduce cavitation risk, whereas *P. balsamifera* maintained turgor and kept stomata open under mild stress, reflecting greater drought tolerance (Groover *et al*., 2025). Differences may also arise from broader geographic sampling in our study, which captured more intraspecific variation than previous studies (Soolanayakanahally *et al*., 2009; Keller *et al*., 2011; Pointeau & Guy, 2014). However, patterns of amphistomy were consistent with previous findings in which *P. balsamifera* generally exhibited none or fewer stomata on the adaxial leaf surface, while *P. trichocarpa* showed greater variation, with more genotypes displaying adaxial stomata (McKown *et al*., 2014, 2019; Fetter *et al*., 2021).

In our study, hybrid genotypes exhibited stomatal traits that were, on average, intermediate between the parental species or biased toward one parent. However, they also spanned a broader range of phenotypic values, indicating that hybridization has generated novel trait combinations that may provide raw material for selection (Hamilton & Miller, 2016; Janes & Hamilton, 2017). Stomatal conductance in hybrids on average was intermediate to *P. balsamifera* and *P. trichocarpa*, consistent with additive genetic contributions from both parental species (Dunning *et al*., 2022). Such additive trait expression may offer functional benefits in transitional environments where hybrids encounter conditions not occupied by either parental species (Janes & Hamilton, 2017). In our study, hybrid genotypes largely resembled *P. trichocarpa* in adaxial stomatal density, occurrence, and stomatal ratio, with the presence of stomata on both leaf surfaces. Previous studies have reported adaxial stomata in *P. balsamifera* admixed genotypes only in regions of hybridization with *P. trichocarpa*, *P. deltoides*, or *P. angustifolia* (Pearce *et al*., 2006; McKown *et al*., 2014, 2019), suggesting that amphistomaty arises through introgression. In hybrids, amphistomaty may provide an advantage, enhancing photosynthetic capacity across transitional environments (Muir, 2019). By contrast, in *P. deltoides × P. angustifolia* hybrids, amphistomatous traits from *P. deltoides* were not retained, and both *P. angustifolia* and hybrids remained largely hypostomatous (Zanewich *et al*., 2018). These contrasting outcomes suggest that the expression of amphistomaty in hybrids may be shaped by differences in parental trait dominance or directional selection (Pfeilsticker *et al*., 2022).

### Climate shapes genetic variation underlying stomatal traits despite high interspecific gene flow

Despite geographic clines indicating limited barriers to gene flow at a trait level across the six latitudinal contact zones, trait variation was strongly associated with climatic gradients, particularly precipitation. Adaxial stomatal density, occurrence, and stomatal ratio were strongly associated with precipitation gradients, although the direction of the relationship varied across several contact zones. In the northern contact zones, wetter origins were associated with higher adaxial stomatal density, with more genotypes with amphistomatous leaves, while drier origins showed reduced adaxial stomata and more genotypes with hypostomatous leaves. This suggests that, in the north, selection may favor amphistomaty under wetter conditions to support rapid growth during short growing seasons, but is selected against in drier climates to limit water loss (McKown *et al*., 2019). Southern contact zones showed the opposite pattern with a lower stomatal ratio for genotypes sourced from wetter environments and higher from drier origins. In the south, selection may favor lower adaxial stomatal density to reduce pathogen entry, reflecting a trade‐off between gas exchange and disease resistance, which is typically higher in warmer, humid regions of the south compared to the north (McKown *et al*., 2014; Fetter *et al*., 2021).

Stomatal size traits, including guard cell and pore length, showed clear associations with precipitation across the hybrid zone. In most contact zones, genotypes from drier environments were linked to smaller abaxial guard cells and shorter pore lengths, supporting the hypothesis that reduced stomatal size reduces water loss under drought stress while allowing faster stomatal closure (Franks & Beerling, 2009; Drake *et al*., 2013; Niemczyk *et al*., 2019; Volk *et al*., 2022). Contrary to expectations that genotypes originating from cooler and drier climates would exhibit reduced stomatal conductance (Chen *et al*., 2024), we observed that these genotypes maintained higher stomatal conductance when evaluated in common gardens. Lower evaporative demand in cooler environments may reduce the cost of water loss, allowing higher stomatal conductance with minimal risk. In drier regions, genotypes may exploit brief water availability by maximizing carbon gain during short favorable periods (Buckley, 2019). However, observed patterns may also reflect environmental differences between the common gardens and the climates of origin (Oubida *et al*., 2015).

### Climate‐driven selection limits gene flow at loci underlying stomatal traits

Admixture mapping identified several candidate genes associated with stomatal traits. The strongest associations were for stomatal ratio, adaxial stomatal density, and occurrence, all linked to a region on chromosome 15. Within this region, we detected the transcription factor *TWIST*, a homolog of *Arabidopsis SPEECHLESS* (*SPCH*). *SPCH* is a well‐established regulator of stomatal development across plant species, including *P. trichocarpa* (Peterson *et al*., 2010; McKown *et al*., 2014, 2019; Song *et al*., 2023). We also identified other candidate genes with putative roles in stomatal regulation and drought response. *TRY* controls adaxial leaf surface trichome patterning (Matías‐Hernández *et al*., 2016), while *AGP22* contributes to cell wall structure through arabinogalactan peptides (Seifert & Roberts, 2007). *NF‐YC10* is a transcription factor that regulates ABA signaling and enhances drought tolerance (Swain *et al*., 2017), and *EPR1* acts as an early phytochrome‐responsive transcription factor (Kuno, 2003). Together, these candidate genes point towards a polygenic architecture underlying stomatal formation and regulation in hybrid poplars, with *SPCH* emerging as a central regulator. Natural allelic variation at *SPCH* has been linked to shifts in stomatal patterning across climatic gradients in *P. trichocarpa*, highlighting its role in local adaptation (McKown *et al*., 2019). Our results suggest that natural variation at this locus also underlies adaptive differentiation across species. As a transcription factor, *SPCH* may act as a molecular switch translating environmental signals into developmental changes (Lau *et al*., 2018), positioning it as a key regulator of stomatal evolution and adaptation to novel climates.

We compared the geographic clines for *SPCH* and other candidate genes to genome‐wide ancestry. Steep localized geographic clines (*w* < 0.04) at *SPCH* and other candidate genes were particularly widest in the northernmost contact zone (Alaska), suggesting broad ecological barriers rather than gene‐specific resistance within this contact zone (Schield *et al*., 2024). One possible explanation is that the sharp transition between wet‐cold coastal and dry‐cold interior climates imposes strong extrinsic selection influencing the distribution of genetic variation within this region. Supporting this, we observed a consistent decline in *P. trichocarpa* ancestry toward drier climates across contact zones (Fig. 5), indicating that precipitation gradients likely play a role in structuring local ancestry at stomatal candidate genes. Similar studies in hybrid zones have shown that precipitation can act as a key selective force influencing patterns of genetic variation and introgression (Hamilton & Aitken, 2013; Menon *et al*., 2021; Zavala‐Paez *et al*., 2025). In contrast to Alaska, a comparison of clines for candidate genes and genome‐wide ancestry in Jasper indicated higher gene flow and limited evidence of reproductive isolation (*w* = 0.23–0.58). Cline centers (*c* = 0.23–0.35) were positioned toward the *P. trichocarpa* range, consistent with asymmetric introgression whereby *P. balsamifera* alleles are moving into *P. trichocarpa* populations at these candidate regions. Differences between Alaska and Jasper may reflect variation in demographic history, hybrid composition, or the strength of selection (McFarlane *et al*., 2024). Nevertheless, consistent climate associations in the Jasper contact zone, particularly with precipitation, suggest that selection is acting, though the strength of selection may be insufficient to completely counteract the homogenizing effects of interspecific gene flow.

By comparison, the Cassiar, Chilcotin, Crowsnest, and Wyoming contact zones exhibited selectively permeable species boundaries, where locus‐specific barriers to introgression were closely shaped by the climate of origin. For example, *SPCH* displayed broader clines in Cassiar (*w* = 0.38) and Chilcotin (*w* = 0.99), but with contrasting centers. In Cassiar, the cline center (*c* = 0.59) was located near the *P. balsamifera* range, suggesting that *P. trichocarpa* alleles are moving into the *P. balsamifera* range. By contrast, the Chilcotin center (*c* = 0.37) fell within the *P. trichocarpa* range, indicating movement of *P. balsamifera* alleles into the distribution of *P. trichocarpa*. Fine‐scale climatic associations were detected in both contact zones (Fig. 5). In Cassiar, *P. trichocarpa* alleles introgress into *P. balsamifera* but are constrained to drier sites, whereas in Chilcotin, *P. balsamifera* alleles introgress into the *P. trichocarpa* range under cool, dry conditions. In the southern contact zones, Crowsnest and Wyoming, *SPCH* exhibited steep clines (*w* = 0.02–0.04), consistent with selection limiting introgression at loci underlying traits with evidence of strong species‐specific adaptive differentiation (Barton & Gale, 1993; Suarez‐Gonzalez *et al*., 2018a,b). These steep clines suggest that barriers to introgression may be particularly strong at the southern edges of the hybrid zone, although limited sampling of *P. balsamifera* in these regions could have contributed to the observed pattern.

Taken together, comparisons across the six latitudinal contact zones reveal that climate‐mediated selection, particularly variation in precipitation, constrains introgression both across the genome and at functionally important candidate genes. Thus, despite widespread hybridization, our findings demonstrate that the environment can maintain divergence at key loci. These results offer insight into the mechanisms that both limit and facilitate the movement of adaptive alleles between species, ultimately shaping the evolution of adaptive traits in hybrid zones.

### Conclusion

This study reveals that stomatal trait evolution in *Populus* hybrid zones is shaped by the interplay of gene flow and climate‐driven selection. Hybrids displayed a wide range of trait values, reflecting admixture between divergent parental species. Variation in stomatal traits was strongly associated with environmental gradients, particularly precipitation, suggesting that water availability plays a central role in influencing trait variation across this hybrid zone despite high levels of gene flow. However, although much of the genome exhibited high levels of interspecific gene flow at a trait scale, several candidate genes associated with amphistomy and stomatal conductance exhibited restricted gene flow. Variation in the movement of genetic variants underlying adaptive traits was largely influenced by the climate of origin, shaping local ancestry distributions. These findings highlight that hybridization can generate novel genetic recombinants and facilitate genetic exchange in areas where barriers to reproduction are limited. But overall, the movement of adaptive genetic variation across species and environmental gradients will be strongly influenced by environmental selection.

## Competing interests

None declared.

## Author contributions

MZ‐P led data collection, research design, data analysis and interpretation, and manuscript writing. SRK contributed to data collection, data analysis and interpretation, and manuscript writing. JH and MCF contributed to data collection and manuscript writing. JAH contributed to data collection, research design, data analysis and interpretation, and manuscript writing.

## Disclaimer

The New Phytologist Foundation remains neutral with regard to jurisdictional claims in maps and in any institutional affiliations.

## Supporting information


**Fig. S1** Comparison of manual and automated stomatal counts using LeafNet.
**Fig. S2** Principal component analysis (PCA) of 25 climate variables associated with genotype origin.
**Fig. S3** Stomatal traits comparison across parental and hybrid genotypes.
**Fig. S4** Relationship between geographic distance and stomatal trait values.
**Fig. S5** Relationship between geographic distance and stomatal trait values 2.
**Fig. S6** Associations between stomatal traits and climatic principal components of genotype origin.
**Fig. S7** Quantile–quantile (QQ) plots of association results for stomatal traits in *Populus* hybrids.
**Fig. S8** Manhattan plots for adaxial and abaxial pore length from admixture mapping analyses.
**Fig. S9** Manhattan plots for abaxial stomatal density and intrinsic water‐use efficiency.
**Fig. S10** Manhattan plots for adaxial and abaxial guard cell length.
**Fig. S11** Manhattan plots for total stomatal density and stomatal conductance.
**Fig. S12** Effect sizes of climate variables on ancestry at stomatal trait candidate genes across six *Populus* hybrid zones.

**Methods S1** Estimating genomic ancestry and geographic clines.

**Notes S1** Putative genomic associations with stomatal trait variation.

**Table S1** Summary of sampling locations, coordinates, and climate variables for each genotype.
**Table S2** ANOVA results for stomatal traits across genotype classes.
**Table S3** Results from linear models testing associations between traits and climatic principal components.
**Table S4** Stomatal trait candidate genes identified through admixture mapping.
**Table S5** Geographic cline parameters for stomatal trait candidate genes.
**Table S6** Logistic regression model results testing associations between local ancestry and climatic gradients.
Please note: Wiley is not responsible for the content or functionality of any Supporting Information supplied by the authors. Any queries (other than missing material) should be directed to the *New Phytologist* Central Office.

## Data Availability

The genomic data analyzed in this study were previously published by Bolte *et al*. (2024) and are available on NCBI under accession number PRJNA996882. All data and scripts used for the analyses are available on Github at: https://github.com/michestzav/Stomata_evolution_Populus.git and the Supporting Information.

## References

[nph70706-bib-0001] Abràmoff MD , Magalhães PJ , Ram SJ . 2004. Image processing with imagej . Biophotonics International 11: 36–41.

[nph70706-bib-0002] Barton NH . 1979. Gene flow past a cline. Heredity 43: 333–339.

[nph70706-bib-0003] Barton NH , Gale KS . 1993. Genetic analysis of hybrid zones. In: Hybrid zones and the evolutionary process. New York, NY, USA: Oxford University Press, 13–45.

[nph70706-bib-0004] Blasini DE , Koepke DF , Grady KC , Allan GJ , Gehring CA , Whitham TG , Cushman SA , Hultine KR . 2021. Adaptive trait syndromes along multiple economic spectra define cold and warm adapted ecotypes in a widely distributed foundation tree species. Journal of Ecology 109: 1298–1318.

[nph70706-bib-0005] Bolte CE , Phannareth T , Zavala‐Paez M , Sutara BN , Can MF , Fitzpatrick MC , Holliday JA , Keller SR , Hamilton JA . 2024. Genomic insights into hybrid zone formation: The role of climate, landscape, and demography in the emergence of a novel hybrid lineage. Molecular Ecology 33: e17430. 38867593 10.1111/mec.17430

[nph70706-bib-0006] Browning BL , Tian X , Zhou Y , Browning SR . 2021. Fast two‐stage phasing of large‐scale sequence data. The American Journal of Human Genetics 108: 1880–1890. 34478634 10.1016/j.ajhg.2021.08.005 PMC8551421

[nph70706-bib-0007] Browning BL , Zhou Y , Browning SR . 2018. A one‐penny imputed genome from next‐generation reference panels. The American Journal of Human Genetics 103: 338–348. 30100085 10.1016/j.ajhg.2018.07.015 PMC6128308

[nph70706-bib-0008] Buckley TN . 2019. How do stomata respond to water status? New Phytologist 224: 21–36. 31069803 10.1111/nph.15899

[nph70706-bib-0009] Buerkle CA , Lexer C . 2008. Admixture as the basis for genetic mapping. Trends in Ecology & Evolution 23: 686–694. 18845358 10.1016/j.tree.2008.07.008

[nph70706-bib-0010] Burke JM , Arnold ML . 2001. Genetics and the fitness of hybrids. Annual Review of Genetics 35: 31–52. 10.1146/annurev.genet.35.102401.085719 11700276

[nph70706-bib-0011] Cai Y , Aihara T , Araki K , Sarmah R , Tsumura Y , Hirota M . 2024. Response of stomatal density and size in *Betula ermanii* to contrasting climate conditions: the contributions of genetic and environmental factors. Ecology and Evolution 14: 1–11. 10.1002/ece3.11349 PMC11184283 38895564

[nph70706-bib-0012] Campbell DR . 2004. Natural selection in Ipomopsis hybrid zones: implications for ecological speciation. New Phytologist 161: 83–90.

[nph70706-bib-0013] Campbell DR , Wendlandt C . 2013. Altered precipitation affects plant hybrids differently than their parental species. American Journal of Botany 100: 1322–1331. 23748678 10.3732/ajb.1200473

[nph70706-bib-0014] Capblancq T , Després L , Mavárez J . 2020. Genetic, morphological and ecological variation across a sharp hybrid zone between two alpine butterfly species. Evolutionary Applications 13: 1435–1450. 32684968 10.1111/eva.12925 PMC7359832

[nph70706-bib-0015] Chen G , Qin Y , Wang J , Li S , Zeng F , Deng F , Chater C , Xu S , Chen ZH . 2024. Stomatal evolution and plant adaptation to future climate. Plant, Cell & Environment 47: 3299–3315. 10.1111/pce.14953 38757448

[nph70706-bib-0016] Chhetri HB , Macaya‐Sanz D , Kainer D , Biswal AK , Evans LM , Chen J , Collins C , Hunt K , Mohanty SS , Rosenstiel T *et al*. 2019. Multitrait genome‐wide association analysis of *Populus trichocarpa* identifies key polymorphisms controlling morphological and physiological traits. New Phytologist 223: 293–309. 30843213 10.1111/nph.15777

[nph70706-bib-0017] Derryberry EP , Derryberry GE , Maley JM , Brumfield RT . 2014. hzar: hybrid zone analysis using an R software package. Molecular Ecology Resources 14: 652–663. 24373504 10.1111/1755-0998.12209

[nph70706-bib-0018] Dias‐Alves T , Mairal J , Blum MGB . 2018. loter: a software package to infer local ancestry for a wide range of species. Molecular Biology and Evolution 35: 2318–2326. 29931083 10.1093/molbev/msy126 PMC6107063

[nph70706-bib-0019] Dittberner H , Korte A , Mettler‐Altmann T , Weber APM , Monroe G , de Meaux J . 2018. Natural variation in stomata size contributes to the local adaptation of water‐use efficiency in *Arabidopsis thaliana* . Molecular Ecology 27: 4052–4065. 30118161 10.1111/mec.14838 PMC7611081

[nph70706-bib-0020] Drake PL , de Boer HJ , Schymanski SJ , Veneklaas EJ . 2019. Two sides to every leaf: water and CO_2_ transport in hypostomatous and amphistomatous leaves. New Phytologist 222: 1179–1187. 30570766 10.1111/nph.15652

[nph70706-bib-0021] Drake PL , Froend RH , Franks PJ . 2013. Smaller, faster stomata: scaling of stomatal size, rate of response, and stomatal conductance. Journal of Experimental Botany 64: 495–505. 23264516 10.1093/jxb/ers347 PMC3542046

[nph70706-bib-0022] Dunning LT , Olofsson JK , Papadopulos AST , Hibdige SGS , Hidalgo O , Leitch IJ , Baleeiro PC , Ntshangase S , Barker N , Jobson RW . 2022. Hybridization and chloroplast capture between distinct *Themeda triandra* lineages in Australia. Molecular Ecology 31: 5846–5860. 36089907 10.1111/mec.16691 PMC9828686

[nph70706-bib-0023] Elfarargi AF , Gilbault E , Döring N , Neto C , Fulgione A , Weber APM , Loudet O , Hancock AM . 2023. Genomic basis of adaptation to a novel precipitation regime. Molecular Biology and Evolution 40: msad031. 36788455 10.1093/molbev/msad031 PMC10037080

[nph70706-bib-0024] Fang Y , Wang D , Xiao L , Quan M , Qi W , Song F , Zhou J , Liu X , Qin S , Du Q *et al*. 2023. Allelic variation in transcription factor *PtoWRKY68* contributes to drought tolerance in *Populus* . Plant Physiology 193: 736–755. 37247391 10.1093/plphys/kiad315 PMC10469405

[nph70706-bib-0025] Farquhar G , O'Leary M , Berry J . 1982. On the relationship between carbon isotope discrimination and the intercellular carbon dioxide concentration in leaves. Functional Plant Biology 9: 121.

[nph70706-bib-0026] Fetter KC , Keller SR . 2023. Admixture mapping and selection scans identify genomic regions associated with stomatal patterning and disease resistance in hybrid poplars. Ecology and Evolution 13: e10579. 37881228 10.1002/ece3.10579 PMC10597741

[nph70706-bib-0027] Fetter KC , Nelson DM , Keller SR . 2021. Growth‐defense trade‐offs masked in unadmixed populations are revealed by hybridization. Evolution 75: 1450–1465. 33914360 10.1111/evo.14227

[nph70706-bib-0028] Franks PJ , Beerling DJ . 2009. Maximum leaf conductance driven by CO_2_ effects on stomatal size and density over geologic time. Proceedings of the National Academy of Sciences, USA 106: 10343–10347. 10.1073/pnas.0904209106 PMC2693183 19506250

[nph70706-bib-0029] Geraldes A , Farzaneh N , Grassa CJ , McKown AD , Guy RD , Mansfield SD , Douglas CJ , Cronk QCB . 2014. Landscape genomics of *Populus trichocarpa*: the role of hybridization, limited gene flow, and natural selection in shaping patterns of population structure. Evolution 68: 3260–3280. 25065449 10.1111/evo.12497

[nph70706-bib-0030] Groover A , Holbrook NM , Polle A , Sala A , Medlyn B , Brodersen C , Pittermann J , Gersony J , Sokołowska K , Bogar L *et al*. 2025. Tree drought physiology: critical research questions and strategies for mitigating climate change effects on forests. New Phytologist 245: 1817–1832. 39690524 10.1111/nph.20326

[nph70706-bib-0031] Hamanishi ET , Thomas BR , Campbell MM . 2012. Drought induces alterations in the stomatal development program in *Populus* . Journal of Experimental Botany 63: 4959–4971. 22760471 10.1093/jxb/ers177 PMC3427991

[nph70706-bib-0032] Hamilton JA , Aitken SN . 2013. Genetic and morphological structure of a spruce hybrid (*Picea sitchensis* × *P. glauca*) zone along a climatic gradient. American Journal of Botany 100: 1651–1662. 23935108 10.3732/ajb.1200654

[nph70706-bib-0033] Hamilton JA , Lexer C , Aitken SN , Hamilton J . 2013. Differential introgression reveals candidate genes for selection across a spruce (*Picea sitchensis × P. glauca*) hybrid zone. New Phytologist 197: 927–938. 23228022 10.1111/nph.12055

[nph70706-bib-0034] Hamilton JA , Miller JM . 2016. Adaptive introgression as a resource for management and genetic conservation in a changing climate. Conservation Biology 30: 33–41. 26096581 10.1111/cobi.12574

[nph70706-bib-0035] Hetherington AM , Woodward FI . 2003. The role of stomata in sensing and driving environmental change. Nature 424: 901–908. 12931178 10.1038/nature01843

[nph70706-bib-0036] Janes JK , Hamilton JA . 2017. Mixing it up: the role of hybridization in forest management and conservation under climate change. Forests 8: 1–16.

[nph70706-bib-0037] Kaluthota S , Pearce DW , Evans LM , Letts MG , Whitham TG , Rood SB . 2015. Higher photosynthetic capacity from higher latitude: foliar characteristics and gas exchange of southern, central and northern populations of *Populus angustifolia* . Tree Physiology 35: 936–948. 26232786 10.1093/treephys/tpv069

[nph70706-bib-0038] Keller SR , Soolanayakanahally RY , Guy RD , Silim SN , Olson MS , Tiffin P . 2011. Climate‐driven local adaptation of ecophysiology and phenology in balsam poplar, *Populus balsamifera* L. (Salicaceae). American Journal of Botany 98: 99–108. 21613088 10.3732/ajb.1000317

[nph70706-bib-0039] Klein MC , Meng Z , Bailey‐Bale J , Milner S , Shi P , Muchero W , Chen J , Tschaplinski TJ , Jacobson D , Lagergren J *et al*. 2025. Climate adaptation in *Populus trichocarpa*: key adaptive loci identified for stomata and leaf traits. New Phytologist 247: 2647–2664. 40728086 10.1111/nph.70343 PMC12371182

[nph70706-bib-0040] Kuno N . 2003. The novel MYB protein EARLY‐PHYTOCHROME‐RESPONSIVE1 is a component of a slave circadian oscillator in Arabidopsis. The Plant Cell Online 15: 2476–2488. 10.1105/tpc.014217 PMC197310 14523250

[nph70706-bib-0041] Lau OS , Song Z , Zhou Z , Davies KA , Chang J , Yang X , Wang S , Lucyshyn D , Tay IHZ , Wigge PA *et al*. 2018. Direct control of SPEECHLESS by PIF4 in the high‐temperature response of stomatal development. Current Biology 28: 1273–1280. 29628371 10.1016/j.cub.2018.02.054 PMC5931714

[nph70706-bib-0042] Li H , Handsaker B , Wysoker A , Fennell T , Ruan J , Homer N , Marth G , Abecasis G , Durbin R . 2009. The sequence alignment/map format and SAMtools. Bioinformatics 25: 2078–2079. 19505943 10.1093/bioinformatics/btp352 PMC2723002

[nph70706-bib-0043] Li L , Jin Z , Huang R , Zhou J , Song F , Yao L , Li P , Lu W , Xiao L , Quan M *et al*. 2023. Leaf physiology variations are modulated by natural variations that underlie stomatal morphology in *Populus* . Plant, Cell & Environment 46: 150–170. 10.1111/pce.14471 36285358

[nph70706-bib-0044] Li S , Li L , Fan W , Ma S , Zhang C , Kim JC , Wang K , Russinova E , Zhu Y , Zhou Y . 2022. LeafNet: a tool for segmenting and quantifying stomata and pavement cells. Plant Cell 34: 1171–1188. 35080620 10.1093/plcell/koac021 PMC8972303

[nph70706-bib-0045] Liu C , Huang K , Zhao Y , Li Y , He N . 2024. A continental‐scale analysis reveals the latitudinal gradient of stomatal density across amphistomatous species: evolutionary history vs present‐day environment. Annals of Botany 134: 877–886. 39136155 10.1093/aob/mcae135 PMC11639198

[nph70706-bib-0046] Liu Q , Wang Z , Yu S , Li W , Zhang M , Yang J , Li D , Yang J , Li C . 2021. Pu‐miR172d regulates stomatal density and water‐use efficiency via targeting PuGTL1 in poplar. Journal of Experimental Botany 72: 1370–1383. 33098429 10.1093/jxb/eraa493

[nph70706-bib-0047] Marek S , Tomaszewski D , Żytkowiak R , Jasińska A , Zadworny M , Boratyńska K , Dering M , Danusevičius D , Oleksyn J , Wyka TP . 2022. Stomatal density in *Pinus sylvestrisas* an indicator of temperature rather than CO_2_: evidence from a pan‐European transect. Plant, Cell & Environment 45: 121–132. 10.1111/pce.14220 34748220

[nph70706-bib-0048] Matías‐Hernández L , Aguilar‐Jaramillo AE , Cigliano RA , Sanseverino W , Pelaz S . 2016. Flowering and trichome development share hormonal and transcription factor regulation. Journal of Experimental Botany 67: 1209–1219. 26685187 10.1093/jxb/erv534

[nph70706-bib-0049] McFarlane SE , Jahner JP , Lindtke D , Buerkle CA , Mandeville EG . 2024. Selection leads to remarkable variability in the outcomes of hybridisation across replicate hybrid zones. Molecular Ecology 33: e17359. 38699787 10.1111/mec.17359

[nph70706-bib-0050] McKown AD , Guy RD , Quamme L , Klápště J , La Mantia J , Constabel CP , El‐Kassaby YA , Hamelin RC , Zifkin M , Azam MS . 2014. Association genetics, geography and ecophysiology link stomatal patterning in *Populus trichocarpa* with carbon gain and disease resistance trade‐offs. Molecular Ecology 23: 5771–5790. 25319679 10.1111/mec.12969

[nph70706-bib-0051] McKown AD , Klápště J , Guy RD , Corea ORA , Fritsche S , Ehlting J , El‐Kassaby YA , Mansfield SD . 2019. A role for *SPEECHLESS* in the integration of leaf stomatal patterning with the growth vs disease trade‐off in poplar. New Phytologist 223: 1888–1903. 31081152 10.1111/nph.15911

[nph70706-bib-0052] Mead A , Beasley‐Bennett JR , Bleich AC , Fischer DG , Flint S , Golightly J , Klopf SK , Kulbaba MW , Lasky J , LeBoldus JM *et al*. 2025. Variation in responses to temperature across admixed genotypes of *Populus trichocarpa* × *P. balsamifera* predict geographic shifts in regions where hybrids are favored. *bioRxiv* . doi: 10.1101/2025.05.16.654548. PMC12780328 41320988

[nph70706-bib-0053] Menon M , Bagley JC , Page GFM , Whipple AV , Schoettle AW , Still CJ , Wehenkel C , Waring KM , Flores‐Renteria L , Cushman SA *et al*. 2021. Adaptive evolution in a conifer hybrid zone is driven by a mosaic of recently introgressed and background genetic variants. Communications Biology 4: 1–14. 33547394 10.1038/s42003-020-01632-7 PMC7864969

[nph70706-bib-0054] Moran ME , Aparecido LMT , Koepke DF , Cooper HF , Doughty CE , Gehring CA , Throop HL , Whitham TG , Allan GJ , Hultine KR . 2023. Limits of thermal and hydrological tolerance in a foundation tree species (*Populus fremontii*) in the desert southwestern United States. New Phytologist 240: 2298–2311. 37680030 10.1111/nph.19247

[nph70706-bib-0055] Muir CD . 2019. Is amphistomy an adaptation to high light? Optimality models of stomatal traits along light gradients. Integrative and Comparative Biology 59: 571–584. 31141118 10.1093/icb/icz085

[nph70706-bib-0056] Niemczyk M , Hu Y , Thomas BR . 2019. Selection of poplar genotypes for adapting to climate change. Forests 10: 1041.

[nph70706-bib-0057] Oubida RW , Gantulga D , Zhang M , Zhou L , Bawa R , Holliday JA . 2015. Partitioning of multivariate phenotypes using regression trees reveals complex patterns of adaptation to climate across the range of black cottonwood (*Populus trichocarpa*). Frontiers in Plant Science 6: 1–12. 25870603 10.3389/fpls.2015.00181 PMC4375981

[nph70706-bib-0058] Pan S , Wang X , Yan Z , Wu J , Guo L , Peng Z , Wu Y , Li J , Wang B , Su Y *et al*. 2024. Leaf stomatal configuration and photosynthetic traits jointly affect leaf water use efficiency in forests along climate gradients. New Phytologist 244: 1250–1262. 39223910 10.1111/nph.20100

[nph70706-bib-0059] Pearce DW , Millard S , Bray DF , Rood SB . 2006. Stomatal characteristics of riparian poplar species in a semi‐arid environment. Tree Physiology 26: 211–218. 16356918 10.1093/treephys/26.2.211

[nph70706-bib-0060] Peterson KM , Rychel AL , Torii KU . 2010. Out of the mouths of plants: the molecular basis of the evolution and diversity of stomatal development. Plant Cell 22: 296–306. 20179138 10.1105/tpc.109.072777 PMC2845417

[nph70706-bib-0061] Pfeilsticker TR , Jones RC , Steane DA , Harrison PA , Vaillancourt RE , Potts BM . 2022. Expansion of the rare *Eucalyptus risdonii* under climate change through hybridization with a closely related species despite hybrid inferiority. Annals of Botany 129: 1–14. 34351372 10.1093/aob/mcab103 PMC8752398

[nph70706-bib-0062] Pointeau VM , Guy RD . 2014. Comparative resource‐use efficiencies and growth of *Populus trichocarpa* and *Populus balsamifera* under glasshouse conditions. Botany 92: 443–451.

[nph70706-bib-0100] R Core Team . 2021. R: A language and environment for statistical computing. R Foundation for Statistical Computing. Vienna, Austria. https://www.R-project.org/

[nph70706-bib-0063] Richardson J , Isebrands JG , Ball JB . 2014. Ecology and physiology of poplars and willows. In: Isebrands J , Richardson J , eds. Poplars and willows: Trees for society and the environment. Oxford, UK: CABI, 92–123.

[nph70706-bib-0064] Rieseberg J , Wendel J . 1993. Introgression and its consequences in plants. In: Futuyma DJ , Shapiro LH , Harrison RG , eds. Hybrid zones and the evolutionary process. New York, NY, USA: Oxford University Press, 110.

[nph70706-bib-0065] Schield DR , Carter JK , Scordato ESC , Levin II , Wilkins MR , Mueller SA , Gompert Z , Nosil P , Wolf JBW , Safran RJ . 2024. Sexual selection promotes reproductive isolation in barn swallows. Science 386: eadj8766. 39666856 10.1126/science.adj8766

[nph70706-bib-0066] Seifert GJ , Roberts K . 2007. The biology of arabinogalactan proteins. Annual Review of Plant Biology 58: 137–161. 10.1146/annurev.arplant.58.032806.103801 17201686

[nph70706-bib-0067] Shriner D , Adeyemo A , Rotimi CN . 2011. Joint ancestry and association testing in admixed individuals. PLoS Computational Biology 7: e1002325. 22216000 10.1371/journal.pcbi.1002325 PMC3245293

[nph70706-bib-0068] Song Z , Wang L , Lee M , Yue GH . 2023. The evolution and expression of stomatal regulators in C_3_ and C_4_ crops: implications on the divergent drought tolerance. Frontiers in Plant Science 14: 1–10. 10.3389/fpls.2023.1100838 PMC9929459 36818875

[nph70706-bib-0069] Soolanayakanahally RY , Guy RD , Silim SN , Drewes EC , Schroeder WR . 2009. Enhanced assimilation rate and water use efficiency with latitude through increased photosynthetic capacity and internal conductance in balsam poplar (*Populus balsamifera* L.). Plant, Cell & Environment 32: 1821–1832. 10.1111/j.1365-3040.2009.02042.x 19712064

[nph70706-bib-0070] Soolanayakanahally RY , Guy RD , Street NR , Robinson KM , Silim SN , Albrectsen BR , Jansson S . 2015. Comparative physiology of allopatric *Populus* species: geographic clines in photosynthesis, height growth, and carbon isotope discrimination in common gardens. Frontiers in Plant Science 6: 1–11. 26236324 10.3389/fpls.2015.00528 PMC4500902

[nph70706-bib-0071] Stankowski S , Sobel JM , Streisfeld MA . 2017. Geographic cline analysis as a tool for studying genome‐wide variation: a case study of pollinator‐mediated divergence in a monkeyflower. Molecular Ecology 26: 107–122. 27065228 10.1111/mec.13645

[nph70706-bib-0072] Suarez‐Gonzalez A , Hefer CA , Lexer C , Douglas CJ , Cronk QCB . 2018a. Introgression from *Populus balsamifera* underlies adaptively significant variation and range boundaries in *P. trichocarpa* . New Phytologist 217: 416–427. 29124769 10.1111/nph.14779

[nph70706-bib-0073] Suarez‐Gonzalez A , Lexer C , Cronk QCB . 2018b. Adaptive introgression: a plant perspective. Biology Letters 14: 20170688. 29540564 10.1098/rsbl.2017.0688 PMC5897607

[nph70706-bib-0074] Swain S , Myers ZA , Siriwardana CL , Holt BF . 2017. The multifaceted roles of NUCLEAR FACTOR‐Y in Arabidopsis thaliana development and stress responses. Biochimica et Biophysica Acta (BBA)–Gene Regulatory Mechanisms 1860: 636–644. 27989935 10.1016/j.bbagrm.2016.10.012

[nph70706-bib-0075] Taylor G . 2002. Populus: Arabidopsis for forestry. do we need a model tree? Annals of Botany 90: 681–689. 12451023 10.1093/aob/mcf255 PMC4240366

[nph70706-bib-0076] Turner DS . 2018. qqman: an R package for visualizing GWAS results using Q–Q and manhattan plots. Journal of Open Source Software 3: 731.

[nph70706-bib-0077] Volk K , Braasch J , Ahlering M , Hamilton JA . 2022. Environmental contributions to the evolution of trait differences in *Geum triflorum*: implications for restoration. American Journal of Botany 109: 1822–1837. 36151780 10.1002/ajb2.16061

[nph70706-bib-0078] Wang T , Hamann A , Spittlehouse D , Carroll C . 2016. Locally downscaled and spatially customizable climate data for historical and future periods for North America. PLoS ONE 11: e0156720. 27275583 10.1371/journal.pone.0156720 PMC4898765

[nph70706-bib-0079] Welch ME , Rieseberg LH . 2002. Habitat divergence between a homoploid hybrid sunflower species, *Helianthus paradoxus* (Asteraceae), and its progenitors. American Journal of Botany 89: 472–478. 21665644 10.3732/ajb.89.3.472

[nph70706-bib-0080] Weng H , Yoo CY , Gosney MJ , Hasegawa PM , Mickelbart MV . 2012. Poplar GTL1 is a Ca^2+^/calmodulin‐binding transcription factor that functions in plant water use efficiency and drought tolerance. PLoS ONE 7: e32925. 22396800 10.1371/journal.pone.0032925 PMC3292583

[nph70706-bib-0081] Whitney KD , Randell RA , Rieseberg LH . 2010. Adaptive introgression of abiotic tolerance traits in the sunflower *Helianthus annuus* . New Phytologist 187: 230–239. 20345635 10.1111/j.1469-8137.2010.03234.x

[nph70706-bib-0082] Wu CA , Campbell DR . 2007. Leaf physiology reflects environmental differences and cytoplasmic background in *Ipomopsis* (Polemoniaceae) hybrids. American Journal of Botany 94: 1804–1812. 21636375 10.3732/ajb.94.11.1804

[nph70706-bib-0083] Zanewich KP , Pearce DW , Rood SB . 2018. Heterosis in poplar involves phenotypic stability: Cottonwood hybrids outperform their parental species at suboptimal temperatures. Tree Physiology 38: 789–800. 29509939 10.1093/treephys/tpy019

[nph70706-bib-0084] Zavala‐Paez M , Sutara B , Keller S , Holliday J , Fitzpatrick MC , Hamilton J . 2025. The role of cytonuclear interactions to plant adaptation across a *Populus* hybrid zone. *bioRxiv* . doi: 10.1101/2025.05.13.653687. PMC12646786 41290169

[nph70706-bib-0085] Zhou X , Stephens M . 2014. Efficient multivariate linear mixed model algorithms for genome‐wide association studies. Nature Methods 11: 407–409. 24531419 10.1038/nmeth.2848 PMC4211878

