# Supplementary material for: Climate and hybridization shape stomatal trait evolution in Populus
Source: New Phytol. 2025 Nov 17;249(2):792–809. doi: 10.1111/nph.70706 (PMC12712426; doi:10.1111/nph.70706)
Supplement: Supplementary file 1 — Fig. S1 Comparison of manual and automated stomatal counts using LeafNet. Fig. S2 Principal component analysis (PCA) of 25 climate variables associated with genotype origin. Fig. S3 Stomatal traits comparison across parental and hybrid genotypes. Fig. S4 Relationship between geographic distance and stomatal trait values. Fig. S5 Relationship between geographic distance and stomatal trait values 2. Fig. S6 Associations between stomatal traits and climatic principal components of genotype origin. Fig. S7 Quantile–quantile (QQ) plots of association results for stomatal traits in Populus hybrids. Fig. S8 Manhattan plots for adaxial and abaxial pore length from admixture mapping analyses. Fig. S9 Manhattan plots for abaxial stomatal density and intrinsic water‐use efficiency. Fig. S10 Manhattan plots for adaxial and abaxial guard cell length. Fig. S11 Manhattan plots for total stomatal density and stomatal conductance. Fig. S12 Effect sizes of climate variables on ancestry at stomatal trait candidate genes across six Populus hybrid zones. [file NPH-249-792-s003.pdf]

New Phytologist Supporting Information

Article title: Climate and hybridization shape stomatal trait evolution in Populus

Authors: Michelle Zavala-Paez, Stephen Keller, Jason Holliday, Matthew C. Fitzpatrick, Jill A. Hamilton

Article acceptance date: 10 October 2025

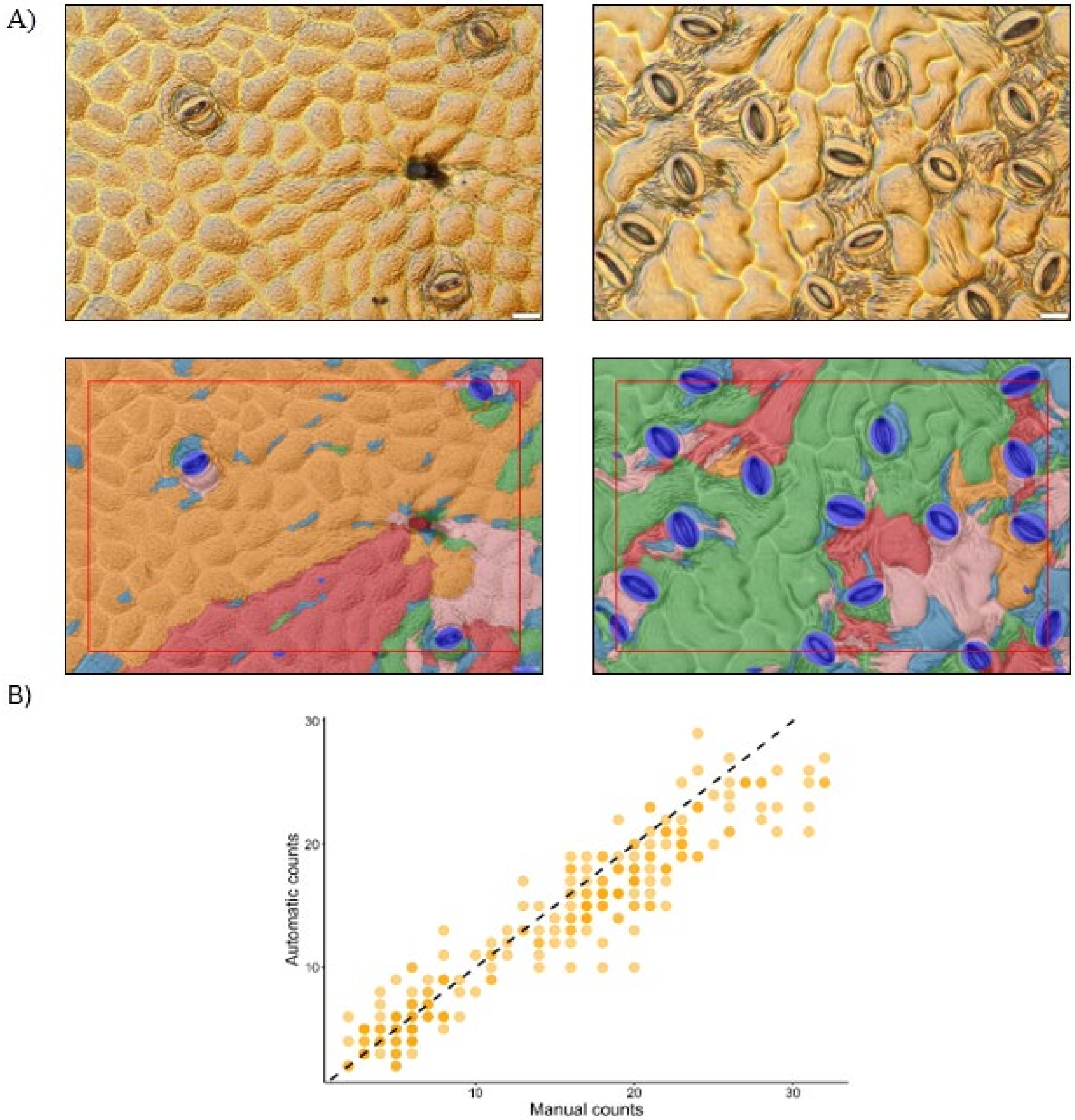

Fig. S1. Comparison of manual and automated stomatal counts using LeafNet. A) Micrographs of epidermal impressions taken at 20 µm scale. LeafNet output highlights detected stomata with blue ovals. B) Spearman correlation ($\rho = 0.95$) between stomatal density estimates obtained manually and using LeafNet, with a 1:1 reference line shown in black.

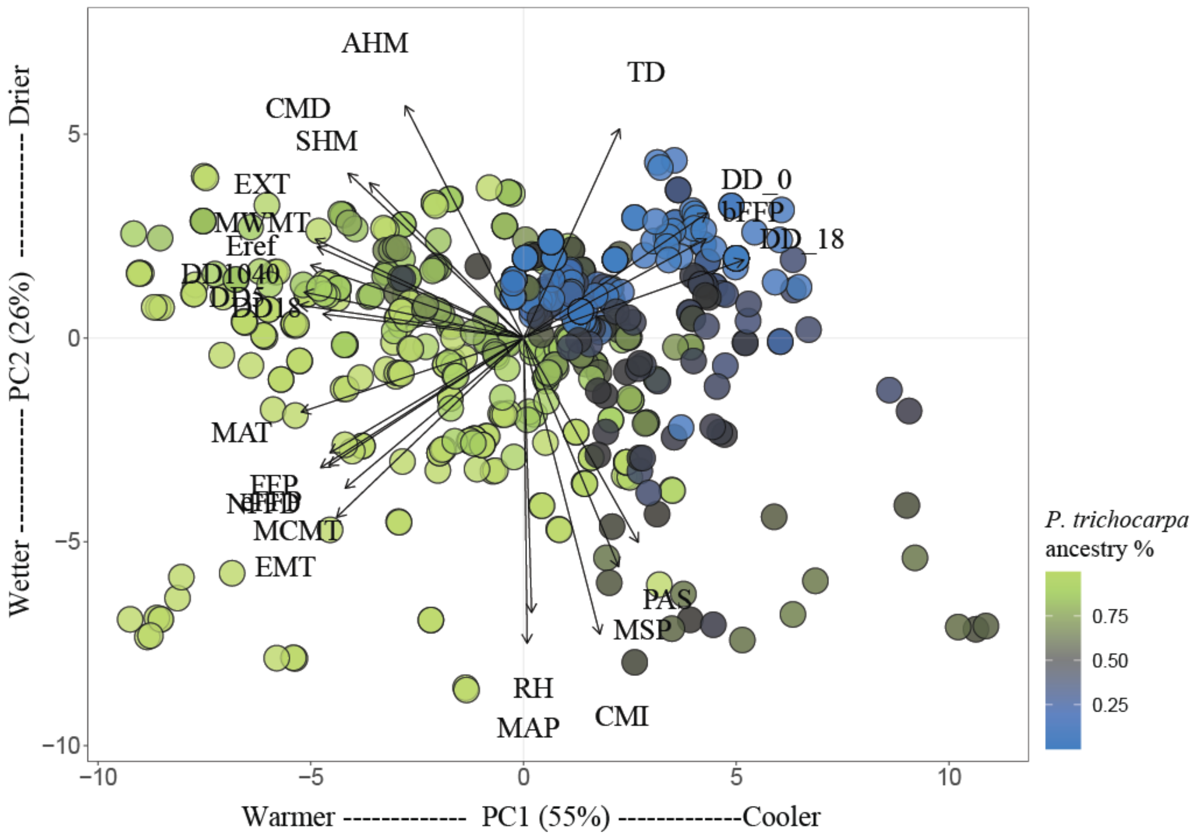

Fig. S2. Principal component analysis (PCA) of 25 climate variables associated with genotype origin. PCA summarizes 25-year climate normals into two major axes. PC1 (55%) captured temperature-related gradients, with high loadings for mean annual temperature and degree-days above 5 °C and 18 °C. PC2 (26%) captured moisture-related gradients, with high loadings for mean annual precipitation, climate moisture index, and relative humidity. Points represent individual sampling sites colored *P. trichocarpa* ancestry variable. Climate vectors indicate variable loadings, with arrows pointing in the direction of increasing values.

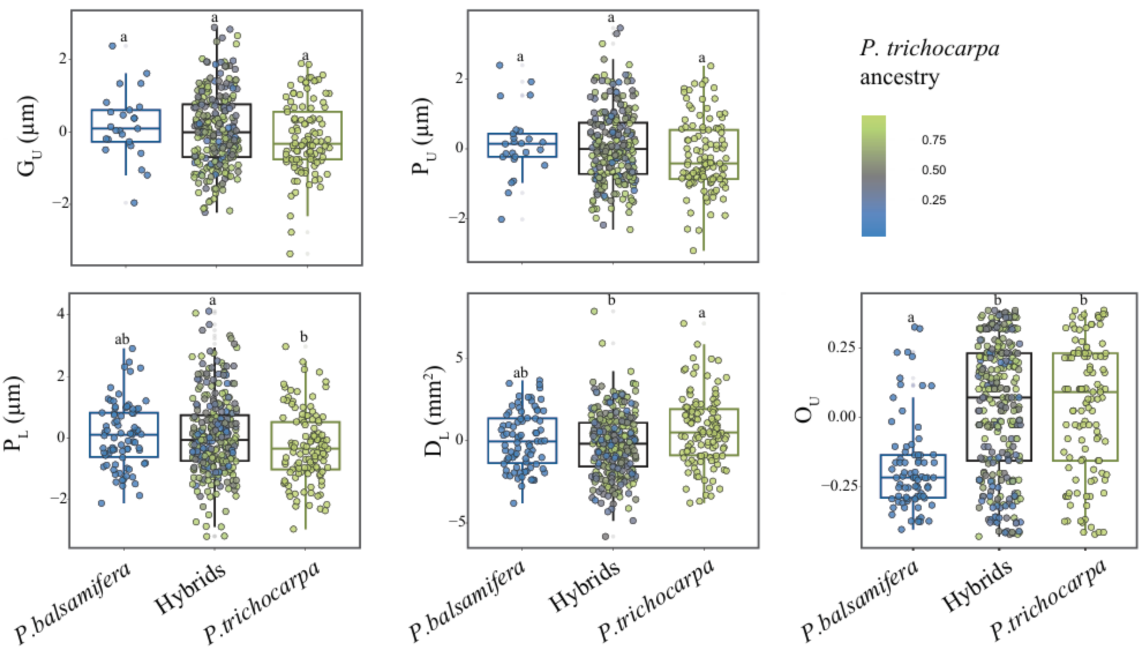

Fig. S3. Stomatal traits comparison across parental and hybrid genotypes. Boxplots show best linear unbiased predictors (BLUPs) for adaxial guard cell length (G_U), adaxial pore length (P_U), abaxial pore length (P_L), abaxial stomatal density (D_L), and abaxial stomatal occurrence (O_U), for *P. balsamifera*, admixed, and *P. trichocarpa* genotypes. Each point represents a genotype, and colors indicate the proportion of *P. trichocarpa* genomic ancestry. Letters denote statistically significant differences among groups based on post-hoc comparisons ($p < 0.05$). The central line represents the median, the box bounds correspond to the interquartile range (IQR; 25th–75th percentiles), whiskers extend to 1.5 × IQR, and points beyond whiskers represent outliers.

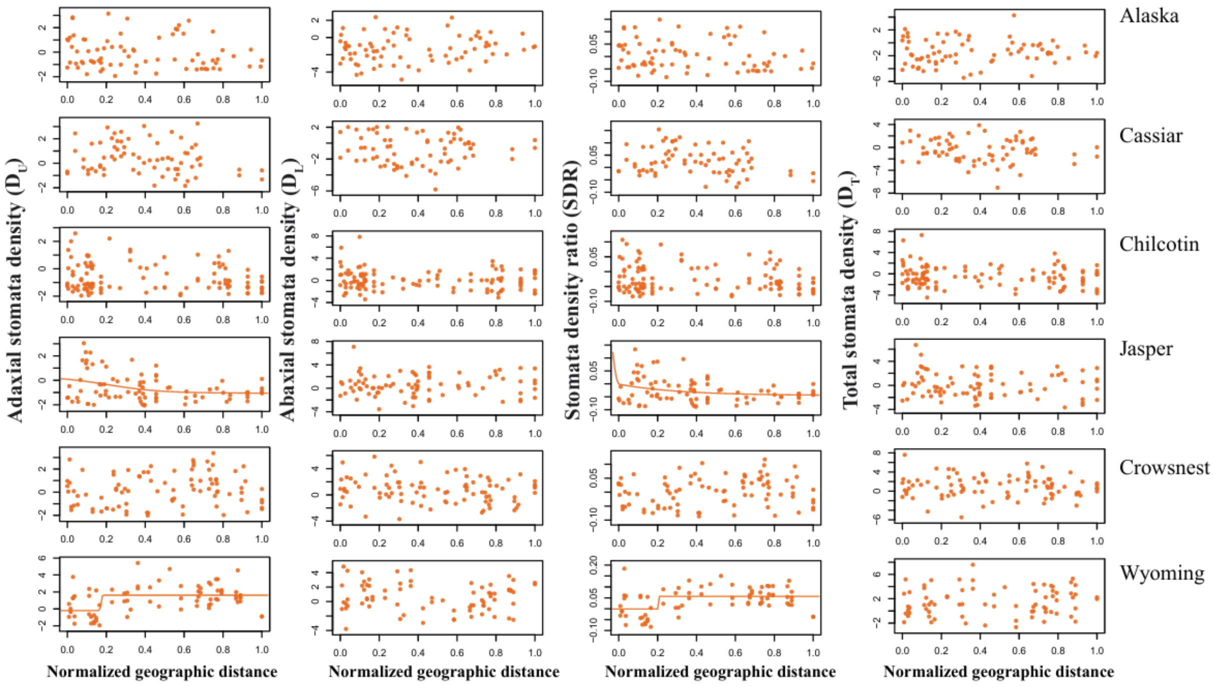

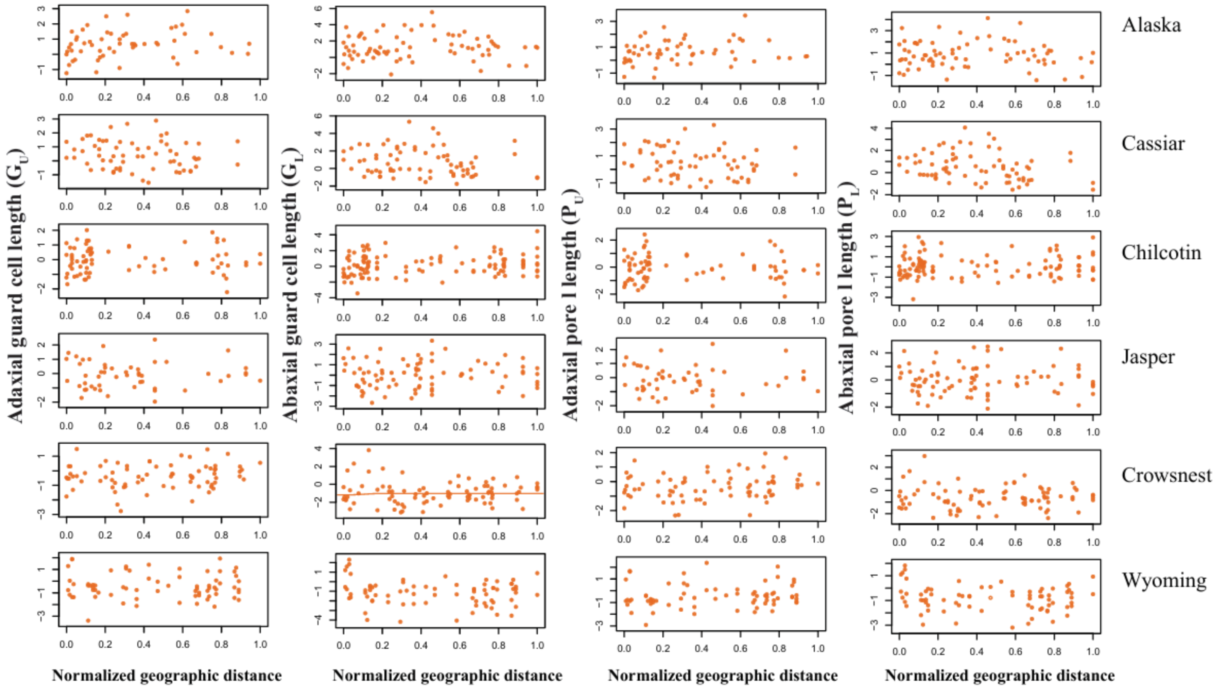

Fig. S4. Relationship between normalized geographic distance and stomatal trait values. Points represent an individual genotype.

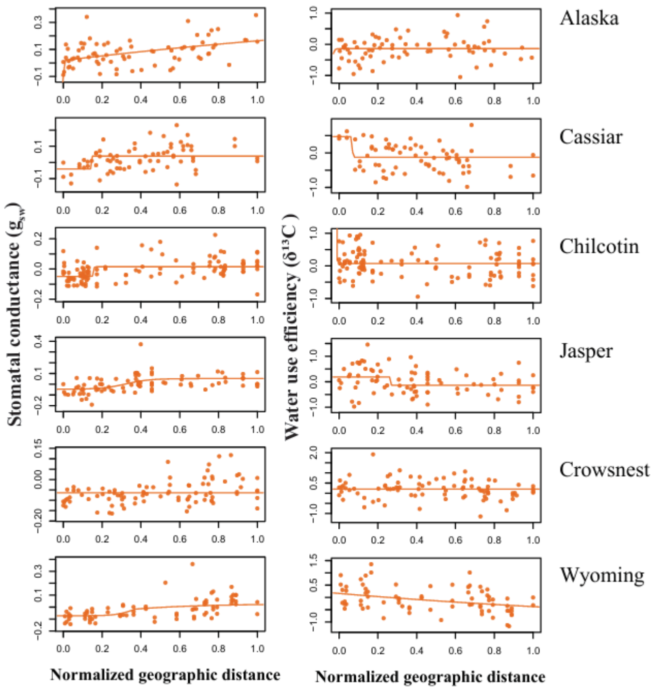

Fig. S5. Relationship between normalized geographic distance and stomatal trait values. Points represent an individual genotype

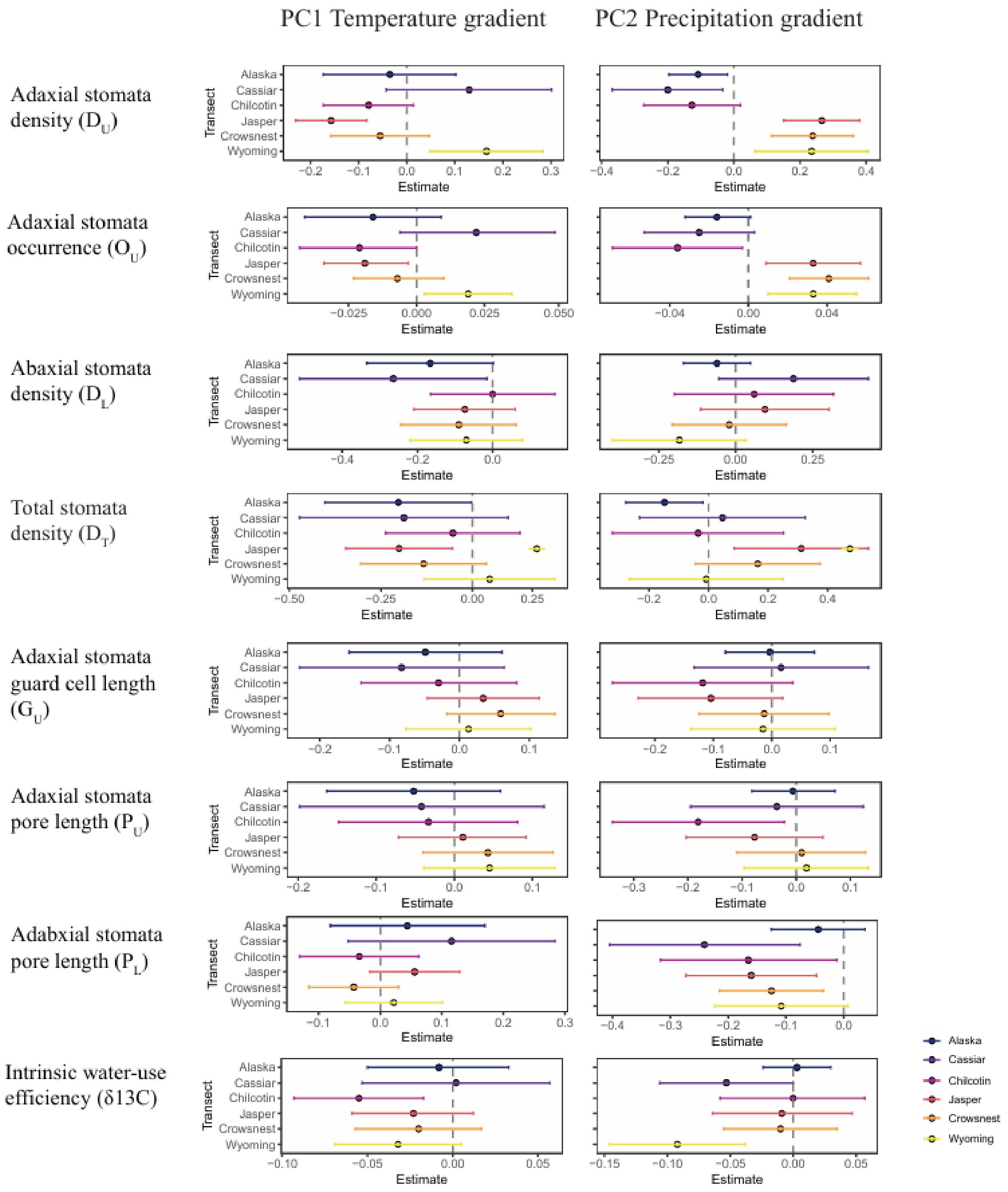

Fig. S6. Associations between stomatal traits and climatic principal components of genotype origin. PC1 reflects a gradient from warmer to cooler environments (positive values = cooler), while PC2 reflects a precipitation gradient (positive values = drier). Panels show trait–transect slopes and their 95 % confidence intervals (CIs), estimated from linear models fitted independently for each transect. Positive slope values indicate trait increases along the climate axis (e.g., toward drier or warmer environments), while negative values reflect decreases.

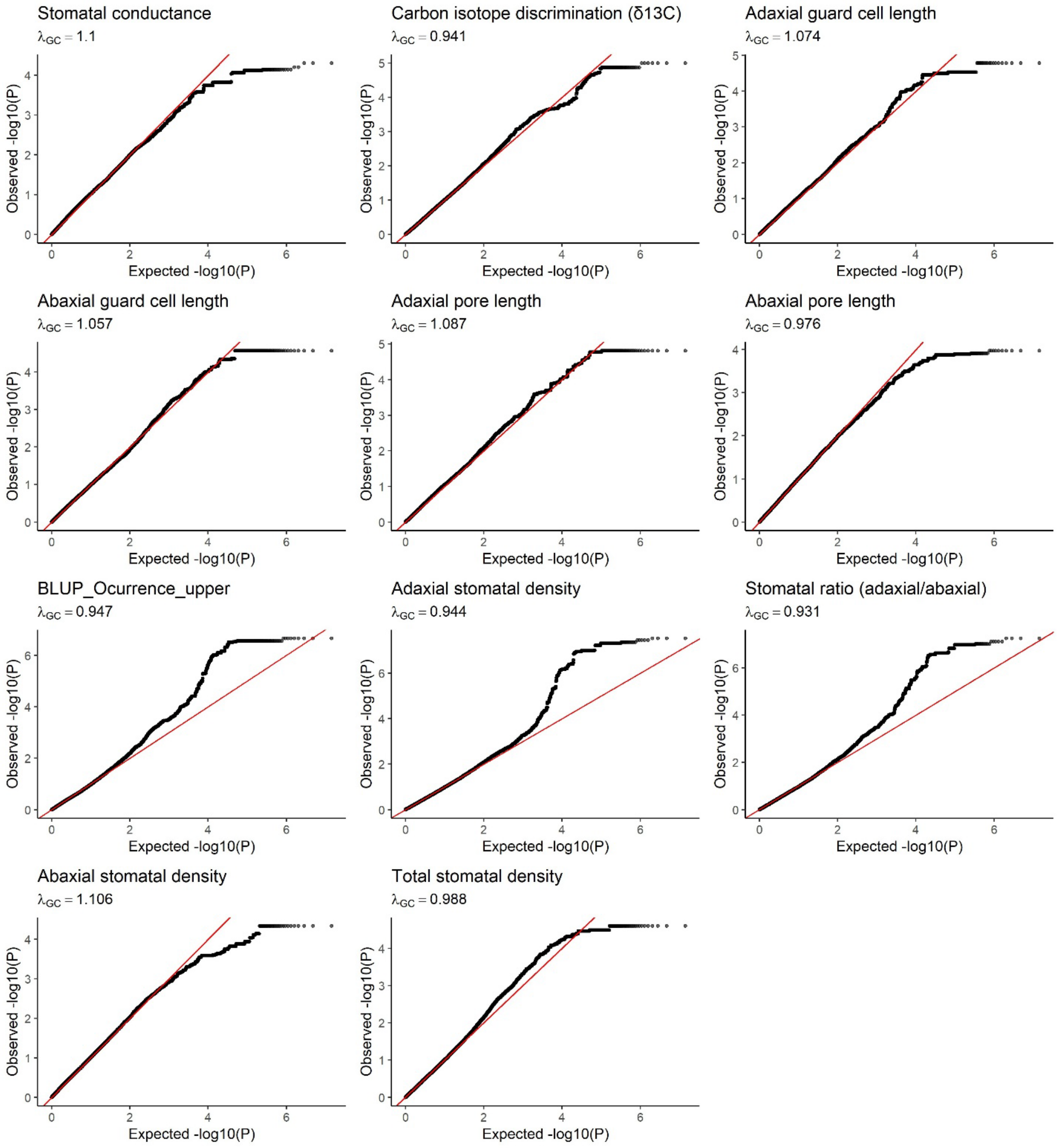

Fig. S7. Quantile–quantile (QQ) plots of association results for stomatal traits in Populus hybrids. Each panel shows the observed versus expected –log10(P) values from GEMMA linear mixed model genome-wide association analyses. Red lines indicate the null expectation under no association. λ_{GC} values denote the genomic control inflation factor for each trait.

Adaxial pore length (P_U)

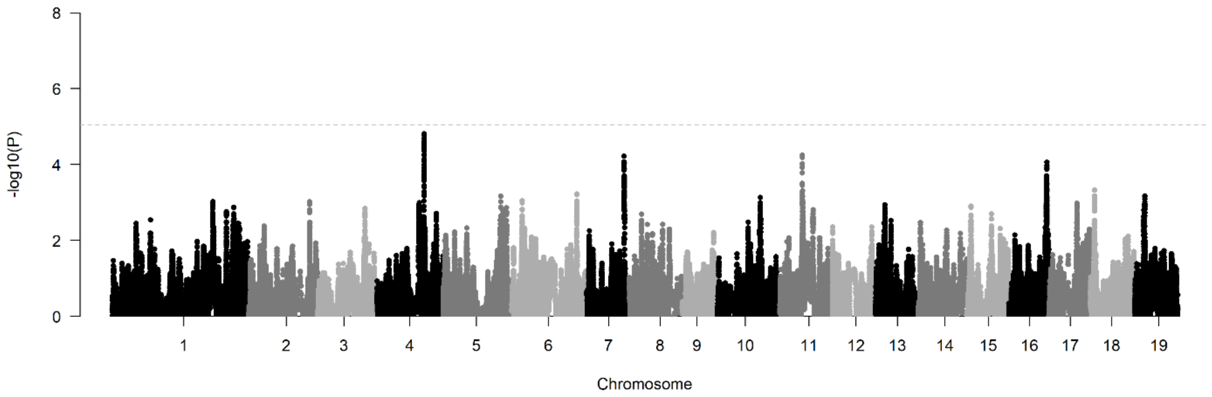

Abaxial pore length (P_L)

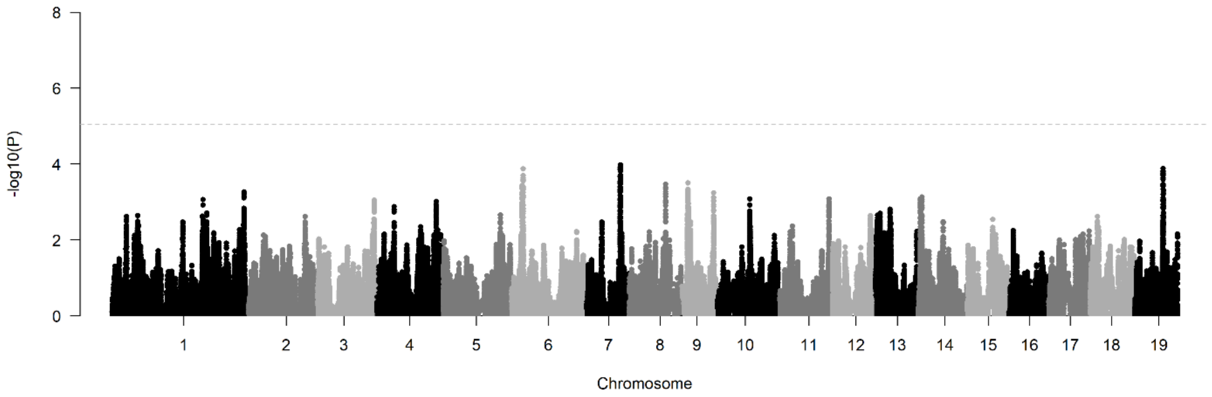

Fig. S8. Manhattan plots showing results from GWAS by admixture mapping for adaxial and abaxial pore length. While no SNPs surpassed the genome-wide significance threshold (gray dashed line; $-\log_{10}(P) \approx 4.73$), several SNPs showed suggestive associations (i.e., below the threshold but potentially meaningful), including some located within genes or within 2 kb upstream

Abaxial stomatal density (D_L)

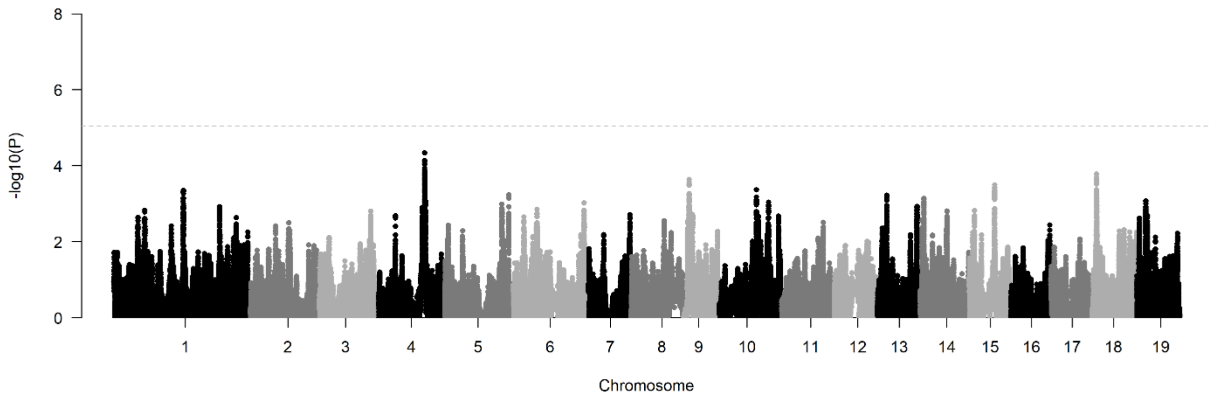

Intrinsic water-use efficiency (δ13C)

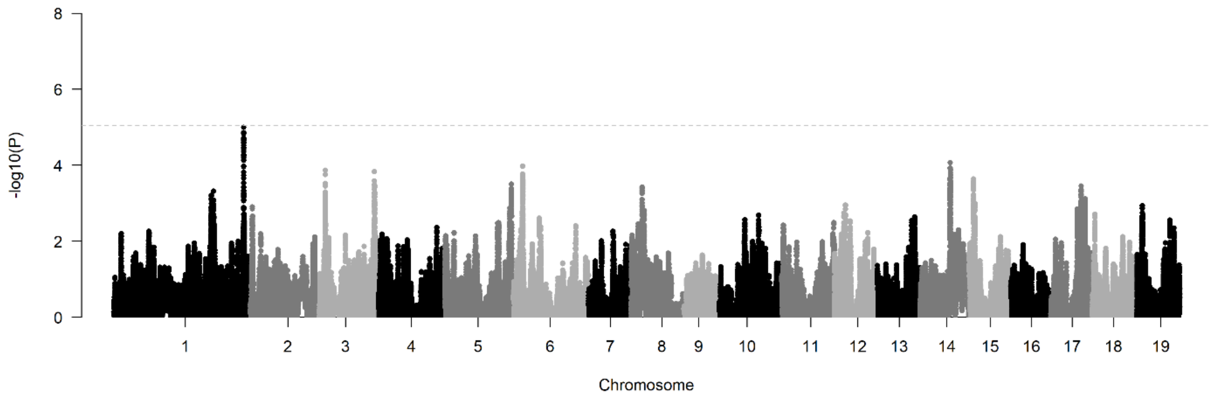

Fig. S9. Manhattan plots showing results from GWAS by admixture mapping for abaxial stomatal density and intrinsic water-use efficiency. While no SNPs surpassed the genome-wide significance threshold (gray dashed line; $-\log_{10}(P) \approx 4.73$), several SNPs showed suggestive associations (i.e., below the threshold but potentially meaningful), including some located within genes or within 2 kb upstream.

Adaxial guard cell length (G_U)

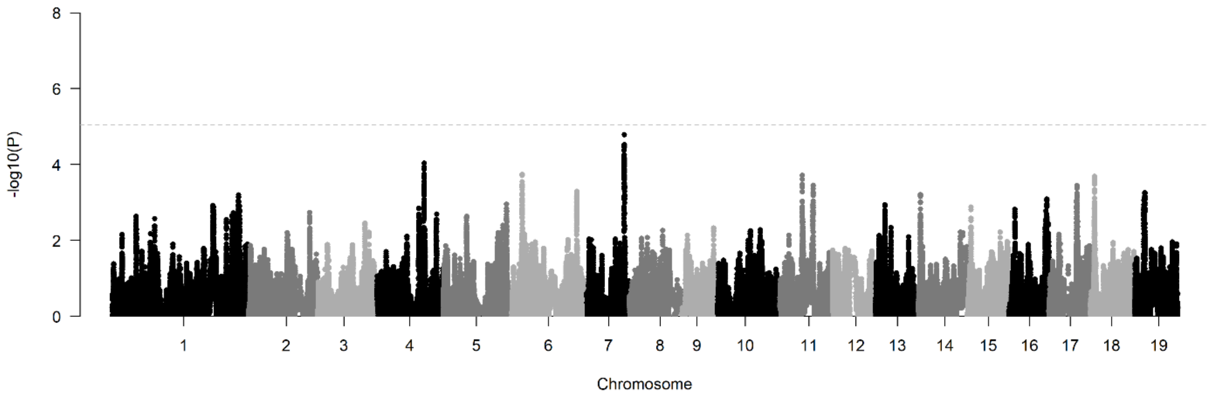

Abaxial guard cell length (G_L)

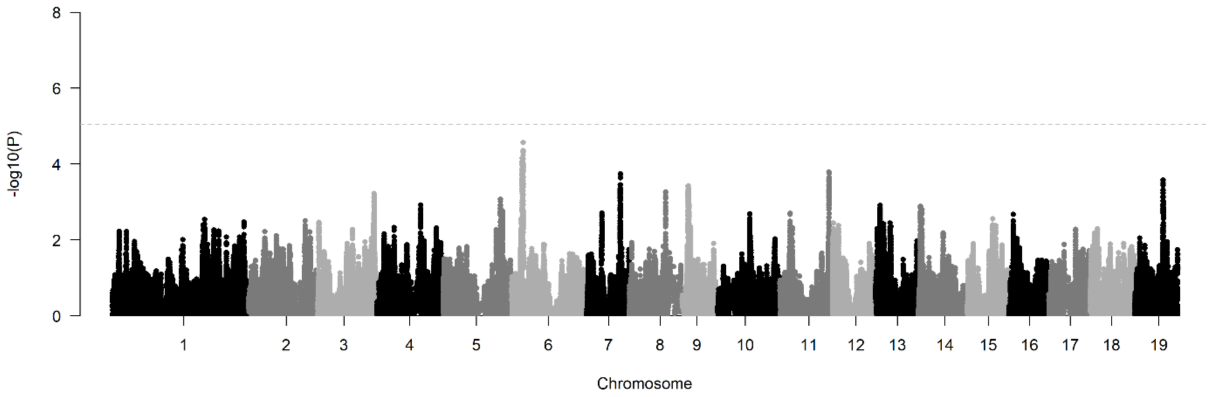

Fig. S10. Manhattan plots showing results from GWAS by admixture mapping for adaxial and abaxial guard cell length. While no SNPs surpassed the genome-wide significance threshold (gray dashed line; $-\log_{10}(P) \approx 4.73$), several SNPs showed suggestive associations (i.e., below the threshold but potentially meaningful), including some located within genes or within 2 kb upstream.

Total stomatal density (D_T)

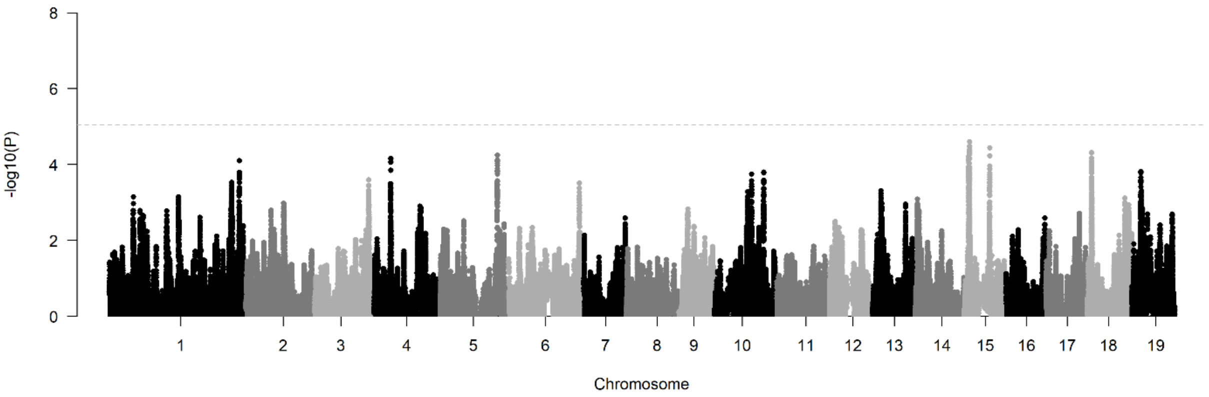

Stomatal conductance (g_{sw})

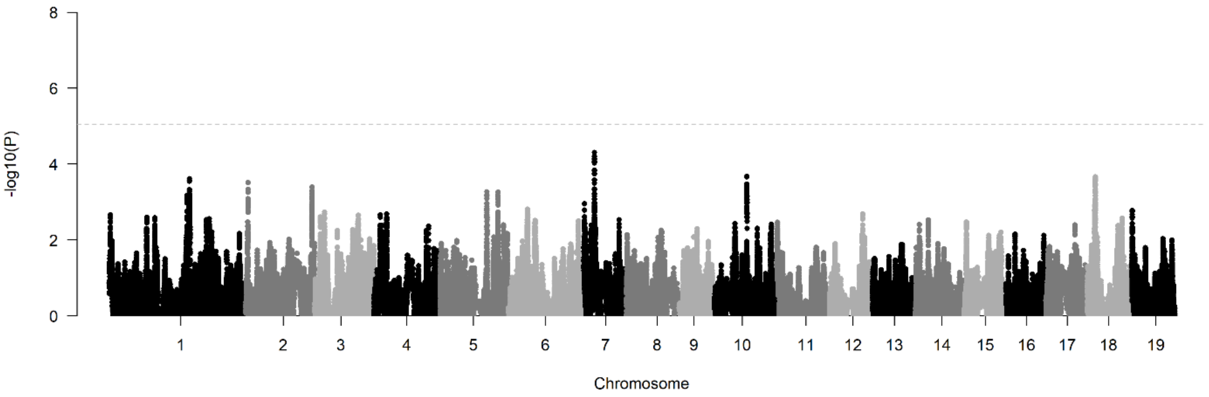

Fig. S11. Manhattan plots showing results from GWAS by admixture mapping for total stomatal density and stomatal conductance. While no SNPs surpassed the genome-wide significance threshold (gray dashed line; $-\log_{10}(P) \approx 4.73$), several SNPs showed suggestive associations (i.e., below the threshold but potentially meaningful), including some located within genes or within 2 kb upstream.

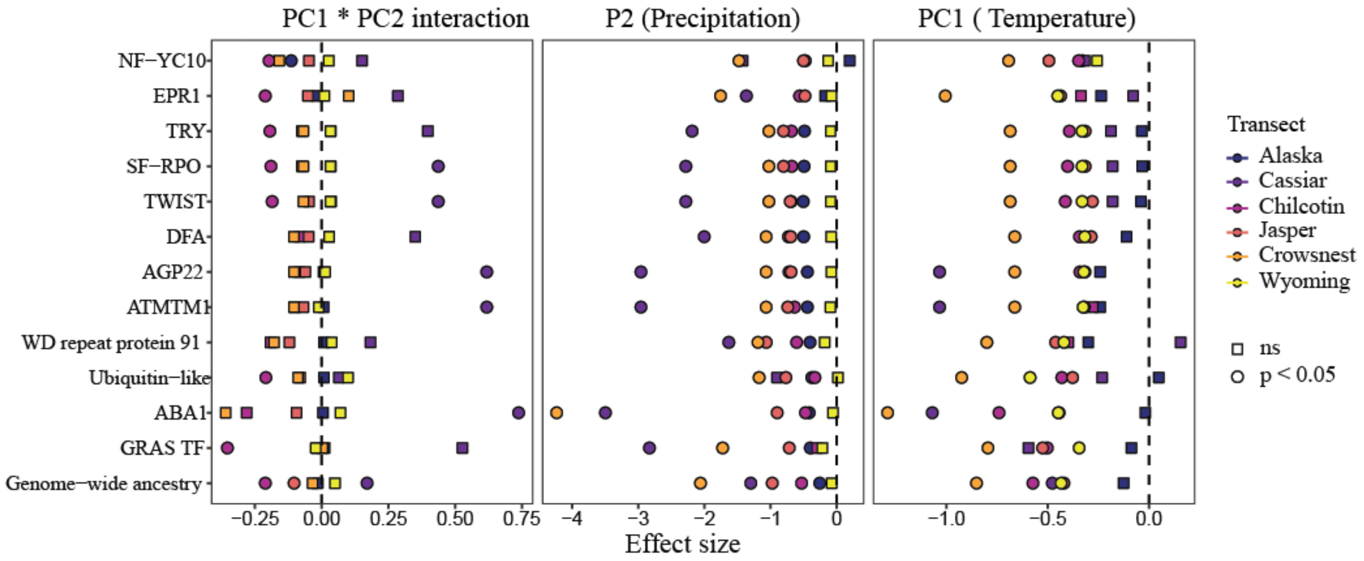

Fig. S12. Effect sizes of climate variables on ancestry at stomatal trait candidate genes across six Populus hybrid zones. Effect sizes from quasibinomial GLMs are shown for the main effects of temperature (PC1), precipitation (PC2), and their interaction (PC1 × PC2). Each point represents the estimated effect for a gene in a specific transect, with fill color indicating transect. Circles indicate statistically significant associations ($p < 0.05$), and squares indicate non-significant associations.
