# Supplementary material for: Climate and hybridization shape stomatal trait evolution in Populus
Source: New Phytol. 2025 Nov 17;249(2):792–809. doi: 10.1111/nph.70706 (PMC12712426; doi:10.1111/nph.70706)
Supplement: Supplementary file 2 — Methods S1 Estimating genomic ancestry and geographic clines. [file NPH-249-792-s002.pdf]

New Phytologist Supporting Information

Article title: Climate and hybridization shape stomatal trait evolution in Populus

Authors: Michelle Zavala-Paez, Stephen Keller, Jason Holliday, Matthew C. Fitzpatrick, Jill A. Hamilton

Article acceptance date: 10 October 2025

Estimating genomic ancestry

To estimate admixture proportions, the VCF file was further filtered using VCFtools to remove SNPs with any missing data across individuals (max-missing = 1.0). Variants were then LD-pruned in PLINK with a 10,000 bp window, shifted by 1,000 bp, removing sites with pairwise $R2$ > 0.1. After filtering and LD-pruning, 334,657 variable sites remained for admixture proportion analysis. Species ancestry across the repeated contact zones was then estimated with ADMIXTURE (K = 2), assigning ancestry proportions to each genotype.

Geographic clines for stomata traits and associated loci

Geographic clines for stomatal traits and associated loci were constructed to test the extent and direction of introgression of the genetic variation underlying stomatal traits. Geographic distances within each contact zone were calculated using the Haversine formula, based on the latitude and longitude coordinates of individual genotypes. For each genotype, geographic distance D_{norm} was normalized using the following equation:

$$D_{norm} = \frac{D_i - D_{min}}{D_{max} + D_{min}}$$

Where: D_i is the geographic distance for genotype i, D_{min} is the minimum geographic distance in the contact zone, and D_{max} is the maximum geographic distance in the contact zone. To facilitate direct comparisons across contact zones, geographic distances were normalized to a scale from 0 to 1.
