# Supplementary material for: Climate and hybridization shape stomatal trait evolution in Populus
Source: New Phytol. 2025 Nov 17;249(2):792–809. doi: 10.1111/nph.70706 (PMC12712426; doi:10.1111/nph.70706)
Supplement: Supplementary file 3 — Notes S1 Putative genomic associations with stomatal trait variation. [file NPH-249-792-s001.pdf]

**New Phytologist Supporting Information**

Article title: Climate and hybridization shape stomatal trait evolution in Populus

Authors: Michelle Zavala-Paez, Stephen Keller, Jason Holliday, Matthew C. Fitzpatrick, Jill A. Hamilton

Article acceptance date: 10 October 2025

**Putative genomic associations with stomatal trait variation**

Admixture mapping analysis showed suggestive associations (i.e., below the threshold but potentially biologically meaningful) for several stomatal traits (Fig. S7-S10). For adaxial guard cell length, a region on chromosome 7 included a *GRAS family transcription factor* (involved in gibberellin-mediated cell elongation). For abaxial guard cell length, a region on chromosome 6 contained Potri.006G059700, encoding a *ubiquitin-like protein*. For total stomatal density, a region on chromosome 15 included *Potri.015G026100*, a *WD repeat-containing protein 91* (involved in protein–protein interactions). Finally, for stomatal conductance, a region on chromosome 7 contained *ABA1*, a key enzyme in abscisic acid biosynthesis that regulates stomatal aperture.
